# A semi-automated material exploration scheme to predict the solubilities of tetraphenylporphyrin derivatives

**DOI:** 10.1038/s42004-022-00770-9

**Published:** 2022-11-22

**Authors:** Raku Shirasawa, Ichiro Takemura, Shinnosuke Hattori, Yuuya Nagata

**Affiliations:** 1 Advanced Research Laboratory, R&D Center, Sony Group Corporation, Atsugi Tec. 4-14-1 Asahi-cho, Atsugi-shi, Kanagawa 243-0014 Japan; 2 Tokyo Laboratory 26, R&D Center, Sony Group Corporation, Atsugi Tec. 4-14-1 Asahi-cho, Atsugi-shi, Kanagawa 243-0014 Japan; 3 Institute for Chemical Reaction Design and Discovery, Hokkaido University, Kita 21 Nishi 10, Kita-ku, Sapporo, Hokkaido 001-0021 Japan

**Keywords:** Cheminformatics, Combinatorial libraries, Optical materials, Spectrophotometry

## Abstract

Acceleration of material discovery has been tackled by informatics and laboratory automation. Here we show a semi-automated material exploration scheme to modelize the solubility of tetraphenylporphyrin derivatives. The scheme involved the following steps: definition of a practical chemical search space, prioritization of molecules in the space using an extended algorithm for submodular function maximization without requiring biased variable selection or pre-existing data, synthesis & automated measurement, and machine-learning model estimation. The optimal evaluation order selected using the algorithm covered several similar molecules (32% of all targeted molecules, whereas that obtained by random sampling and uncertainty sampling was ~7% and ~4%, respectively) with a small number of evaluations (10 molecules: 0.13% of all targeted molecules). The derived binary classification models predicted ‘good solvents’ with an accuracy >0.8. Overall, we confirmed the effectivity of the proposed semi-automated scheme in early-stage material search projects for accelerating a wider range of material research.

## Abstract (web summary)

Prediction of material properties is crucial for early stages of material research, but current experimental data-based strategies possess limited accuracy. Here, the authors develop a machine learning-based semi-automated material exploration scheme to predict the solubility of tetraphenylporphyrin derivatives with an accuracy above 0.8.

## Introduction

Based on the recent advancements in computers and automation, automated experiment schemes have been extensively studied for the discovery or development of novel materials^1–5^. Generally, these schemes can be implemented as actual systems to integrate automation equipment for synthesis and measurement, such as machine-learning (ML) algorithms and controlling software, for the search of novel materials or optimization of the syntheses processes with hardly any human intervention. The application range of these systems is expanding from the search of biologically active materials^6,7^ to the synthesis of coordination compounds^8^, growth of carbon nanotubes^9^, development of materials for clean energy^10^, and the search and optimization of new reactions^11–14^.

### Solubility of Tetraphenylporphyrin

**Fig. 1:**
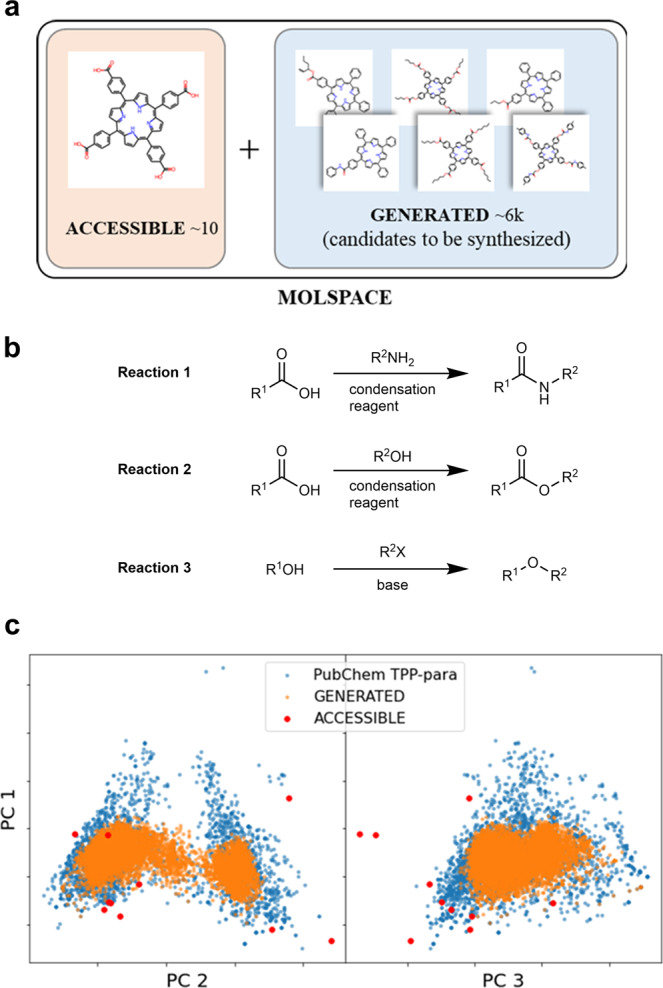
Our target chemical space of TPP derivatives. **a** MOLSPACE contains ACCESSIBLE (*n* = ~10) and GENERATED (*n* = ~6k). **b** GENERATED molecules are generated by three simple reactions: Reaction 1; amid formation, reaction 2; esterification, and reaction 3; Williamson ether synthesis. **c** Mapping molecular groups over principal components (PC 1–3; contribution rate: 0.74) for all molecules with tetraphenylporphyrin (TPP) in PubChem. TPP-para group includes any TPP derivatives with at least one para-substitution to any phenyl group referred from PubChem.

Tetraphenylporphyrin (TPP) derivatives are essential for various applications such as photothermal therapy (PTT) and photodynamic therapy (PDT)^15–17^, dye-sensitized solar cells^18–20^, and photoconductors^21–23^. Thus, extensive research has been conducted to improve or optimize their properties. However, to the best of our knowledge, the method of automated experimental schemes has not yet been applied for molecules with a size comparable to that of TPP. In the syntheses of TPPs, the prediction of their solvent-solubilities for the selection of appropriate solvents is a critical task, because freshly synthesized TPPs are frequently insoluble or form aggregates under certain conditions^24–27^. As the optical properties of the synthesized molecules are significantly impacted in insoluble solvents or aggregated forms, the factors of their performance cannot be conveniently determined to assess their potential practical applicability. In addition, the solubility and aggregation properties of the porphyrins have a significant impact on the properties of their excited state (ES), which directly affect the performance of the porphyrins for the dyes of PTT and PDT^28^. Moreover, in natural light-harvesting system, the electronic excitations of porphyrins are considered to be efficiently transferred by the existence of their aggregates^29–33^. Although infinite variations of the TPP derivatives could be considered, we practically focused on a ‘subspace’ to reduce the total number of such variations with structural constraints (Fig. 1a). In particular, ACCESSIBLE contains commercially available TPP derivatives and certain similar molecules. GENERATED is a computationally generated list of the TPP derivatives, which can be obtained from a simple one-step synthesis of ACCESSIBLE and commercially available small substituents (MW < 200). Furthermore, we considered three simple synthetic processes to prepare the list: amide formation, esterification, and Williamson ether synthesis (Fig. 1b, reactions 1–3). The list of substitution target molecules from ACCESSIBLE is presented in Supplementary Fig. 1, and the list of all substituents is provided in Supplementary Data 1. The mappings of the ACCESSIBLE and GENERATED molecules over currently known TPP derivatives (para-substitution) are plotted in Fig. 1c, excluding the metals extracted from the PubChem—the largest database for organic molecules^34^. In the figure, the axes represent three principal components (PC1–3) depicting the Tanimoto distances in the ECFP6 molecular fingerprint, wherein each molecule is expressed as a series of binary digits and each digit denotes a maximally six-covalent-bond-length substructure^35,36^ (see Supplementary Figs. 2–4 for details). Although the GENERATED list had obvious biases, e.g., the reactions with no large substituents (MW $\ge$ 200) generate limited molecular kinds (amide, ester, and ether) overlapping with only the subspace of all existing TPP derivatives, it still covered a large portion of the dataset. Those limitations will be addressed by introducing other synthetic processes, e.g., Suzuki coupling with larger substituents. The complete list of ACCESSIBLE and GENERATED is provided in Supplementary Data 1, and sampled list of uncovered PubChem’s molecules is presented in Supplementary Fig. 5.

Additionally, automated experimental schemes have been successfully implemented for material searches or the process optimization of ‘well-defined’ target properties, i.e., evidently measured as continuous or categorical values^37,38^. For instance, synthetic yields can be optimized if they are automatically and appropriately measured using high-performance liquid chromatography, and the biological activities of molecules can be detected using luminescent probes. However, the characterization of properties in several material science projects is complex, especially the incomprehensibility of the measured data describing multiple physical effects^39,40^. In this research, we employed ultraviolet–visible light (UV–Vis) absorption spectroscopy to analyze the solubility of TPP derivatives and validate the application of automated experiment schemes for such problems with complicated measurements. Although a UV–Vis absorption spectrum appears simple, it bears complexity because the spectrum is affected by the light-absorption of a single dye as well as the influences of solvent and dye-forming aggregation^41–44^. Thus, from each spectrum, we selected four well-defined values that were adequately informative for representing these physical effects of solutions. Based on these values, we analyzed the solubility and estimated both the qualitative and quantitative models. Additional indicators such as the n-octanol/water partition coefficient (log P)^45^ or Hansen solubility parameters and its expansion^46,47^, which are directly evaluated from single molecular structures based on empirical and theoretical models, can provide insights for determining the solubility. Furthermore, although there have existed recent attempts to predict solubility of organic molecules in medical chemistry^48–50^, solubility prediction of functional materials including organic semiconductor molecules is still limited. Therefore, the solubility of a specific molecular family should be characterized, especially for relatively large and less-soluble molecules such as TPP derivatives (molecular weight (MW) >600), as targeted in this research. Thus, we derived ML models to predict the solubility of practical TPP derivatives and clarified their key factors by utilizing four well-defined indicators evaluated from the UV–Vis absorption spectra obtained from the automated experimentations.

### Fair dataset

Researchers in the fields of material science and chemistry utilize these automated experiment schemes to generate comparable and reproducible data in adequate quantity with appropriate quality^51,52^. This aspect is specifically important for data-driven materials science projects, wherein the experimental data acquired from various sources (literature or database) tend to be limited in quantity and could include errors or inconsistencies that may misdirect the ML model estimations and analyses. In particular, these errors or inconsistencies can result from several reasons such as human errors, environmental effects (atmospheric pressure, temperature, humidity, etc.), experimental parameters (concentration, measuring duration, solution temperature, etc.), various instrument settings, and impurities. For instance, UV–Vis spectra vary with solvents, concentrations, or measuring processes^53,54^, and thus, the direct comparison of these data could produce errors. Currently, researchers are accepting the findable, accessible, interoperable, and reusable (FAIR) data principles to gradually improve the situation^55^. In addition to the errors or inconsistencies, the unavailability of failure data in the existing data sources is critical. In practice, a considerable number of invalid points exist in the targeted chemical space defined by the material structural variables or process parameters, which are actually unmeasurable and yield no data. These failure data are scantily provided by the existing data sources^56^. Thus, in this research, we set up an automated synthesis equipment connected with a UV–Vis absorption spectrometer to obtain a truly FAIR dataset combined with the failure data, which did not exist earlier and can be considered suitable for solubility analysis.

### Selection algorithm

Additionally, automated experiment schemes are advantageous, because the algorithm enables rational experimental planning that avoids insufficient repetitions or local stacks in the chemical spaces^57–60^. Without an algorithm, researchers tend to search molecules with familiar structures. In this context, Bayesian optimization (BO) or its extensions such as Phoenics and Gryffin have been successfully applied to reduce the number of iterations of these automated experiments. As the function of BOs is restricted to input variables with intrinsic low dimensionalities, researchers must reduce the number of variables using certain techniques^61–63^. However, in general, the number of variables is not facilely reduced in the initial stages of material research, because the essential variables remain unidentified, and therefore, the BOs cannot be effectively applied. In the research stage, scholars must comprehend the behavior of materials across the entire chemical space to decide the target of material development along with the essential variables. Generally, these decisions are implicitly conducted based on the researchers’ experiences or intuitions, which can be easily biased. Therefore, to effectively apply the automation schemes in the initial stages of material search, this study implemented an algorithm categorized as submodular function maximization (SFM) to search a subspace yielding the most suitable performance in coverage of chemical space and priorities the materials for a given number of selections from all candidates^64,65^. The coverage of chemical space is an essential indicator that describes the progress of material exploration or screening^66,67^. With the utilization of the SFM algorithm, the present automated experiment system effectively searched a broader space in MOLSPACE within fewer iterations without any biased selection of variables.

**Fig. 2:**
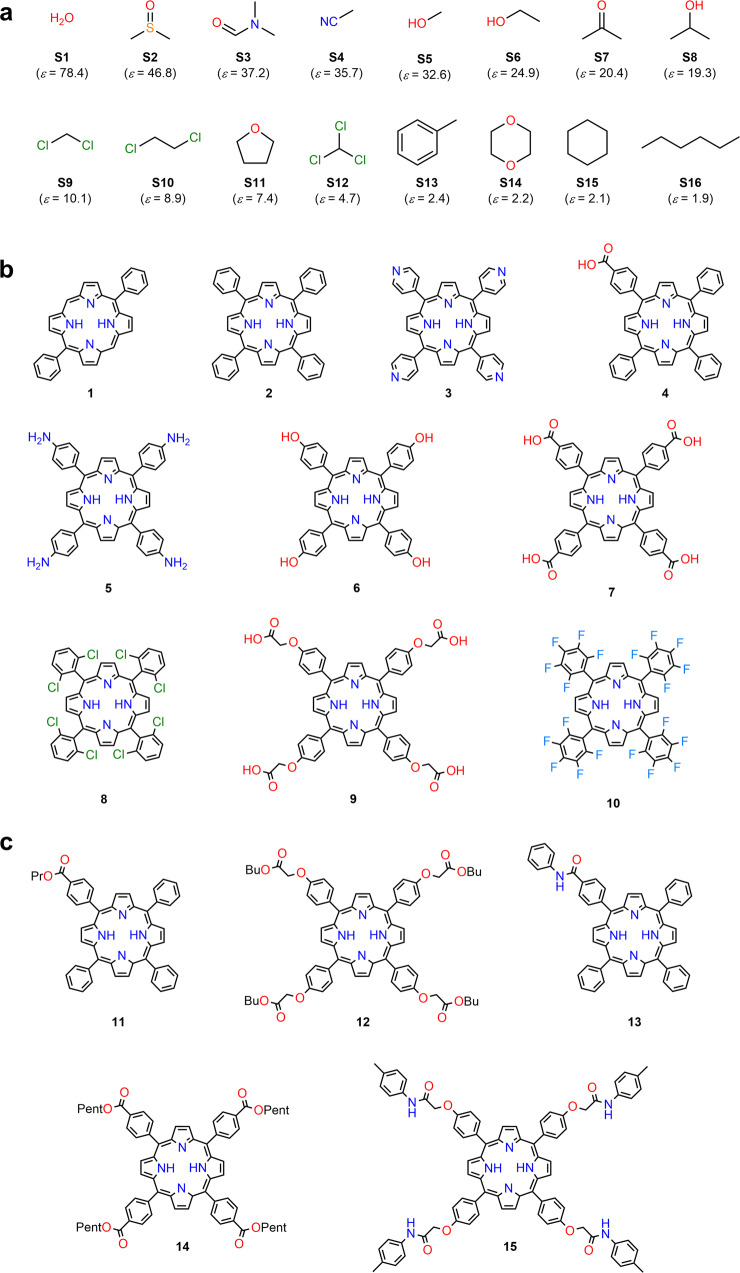
Structures of solvents and TPP derivatives in this study. **a** Solvent list with dielectric constant (*ε*) considered from values for solvent model (SCRF) of Gaussian 16^79^: **S1**: water, **S2**: dimethyl sulfoxide, **S3**: *N*,*N*-dimethylformamide, **S4**: acetonitrile, **S5**: methanol, **S6**: ethanol, **S7**: acetone, **S8**: 2-propanol, **S9**: dichloromethane, **S10**: dichloroethane, **S11**: tetrahydrofuran, **S12**: chloroform, **S13**: toluene, **S14**: 1,4-dioxane, **S15**: cyclohexane, and **S16**: *n*-hexane. **b** Chemical structures of molecules in ACCESSIBLE (**1**–**10**). **c** Five synthesized TPP derivatives with highest priorities from GENERATED (**11**–**15**). For GENERATED, molecules are listed in order of priority (**11** is highest priority). All 15 molecules (**1**–**15**) covered 24.8% of MOLSPACE.

This research aims to develop practical ML models to predict the solubility of TPP derivatives and clarify their key factors based on the current automated experiment scheme. Accordingly, we employed a Chemspeed SWING robotic system connected with UV–Vis absorption spectroscopy (JASCO V-730) and integrated the developed SFM algorithm with the ML models to implement a total analysis scheme for the solubility of TPP derivatives. In total, we selected 16 solvents to cover a wide range of the dielectric constants (*ε*)—from *n*-hexane (*ε* = 1.88) to water (*ε* = 78.4) (Fig. 2a). The sample size of 16 solvents was selected to fit the column size of the current automated experiment equipment. Furthermore, the advantages and limitations of the proposed method are discussed herein.

## Results

### Implementation of automated experiment scheme for solubility analysis

**Fig. 3:**
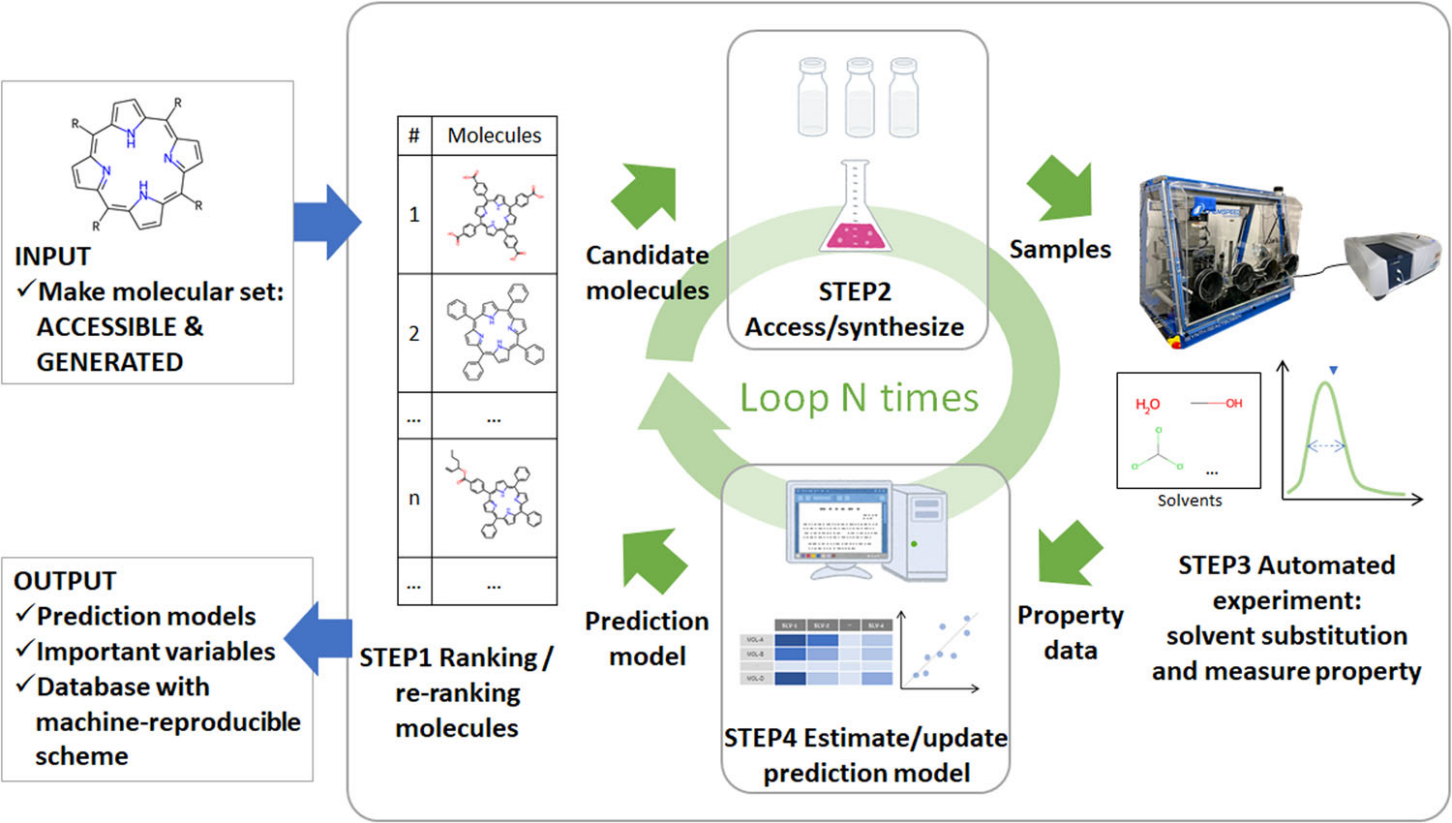
Semi-automated scheme. STEP1; the priority ranking of the INPUT molecular set was constructed using the SFM algorithm. STEP2; a molecule with the highest priority was accessed or synthesized to prepare a solution for measurement in the following step. STEP3; the system performed UV–Vis absorption spectroscopy to observe the spectrum. STEP4; the system evaluated the four representative indicators from each spectrum and estimated both the qualitative and quantitative prediction models based on the accumulated data.

The schematic of the automated experiment system including the four steps implemented in this research is illustrated in Fig. 3. In the first step (STEP1), the priority ranking of the INPUT molecular set was constructed using the SFM algorithm. In the subsequent step (STEP2), a molecule with the highest priority was accessed (if available) or synthesized to prepare a solution for measurement in the following step. In STEP3, the system performed UV–Vis absorption spectroscopy to observe the spectrum of the molecule in all 16 solvents. Lastly, in STEP4, the system evaluated the four representative indicators from each spectrum and estimated both the qualitative and quantitative prediction models based on the accumulated data. The system outputs contributed toward the UV–Vis absorption spectra data, reproducible measurement process (as Chemspeed project), and ML models. In addition to the prediction models, we used the random forest method to highlight the importance of the input variables^68^. To ensure solubility analyses for a diverse range of organic molecules, STEP2 and STEP3 were performed using the current experimental instruments, whereas STEP1 and STEP4 were executed by the Python script written on a Linux PC.

### Generation and prioritization of molecules

**Fig. 4:**
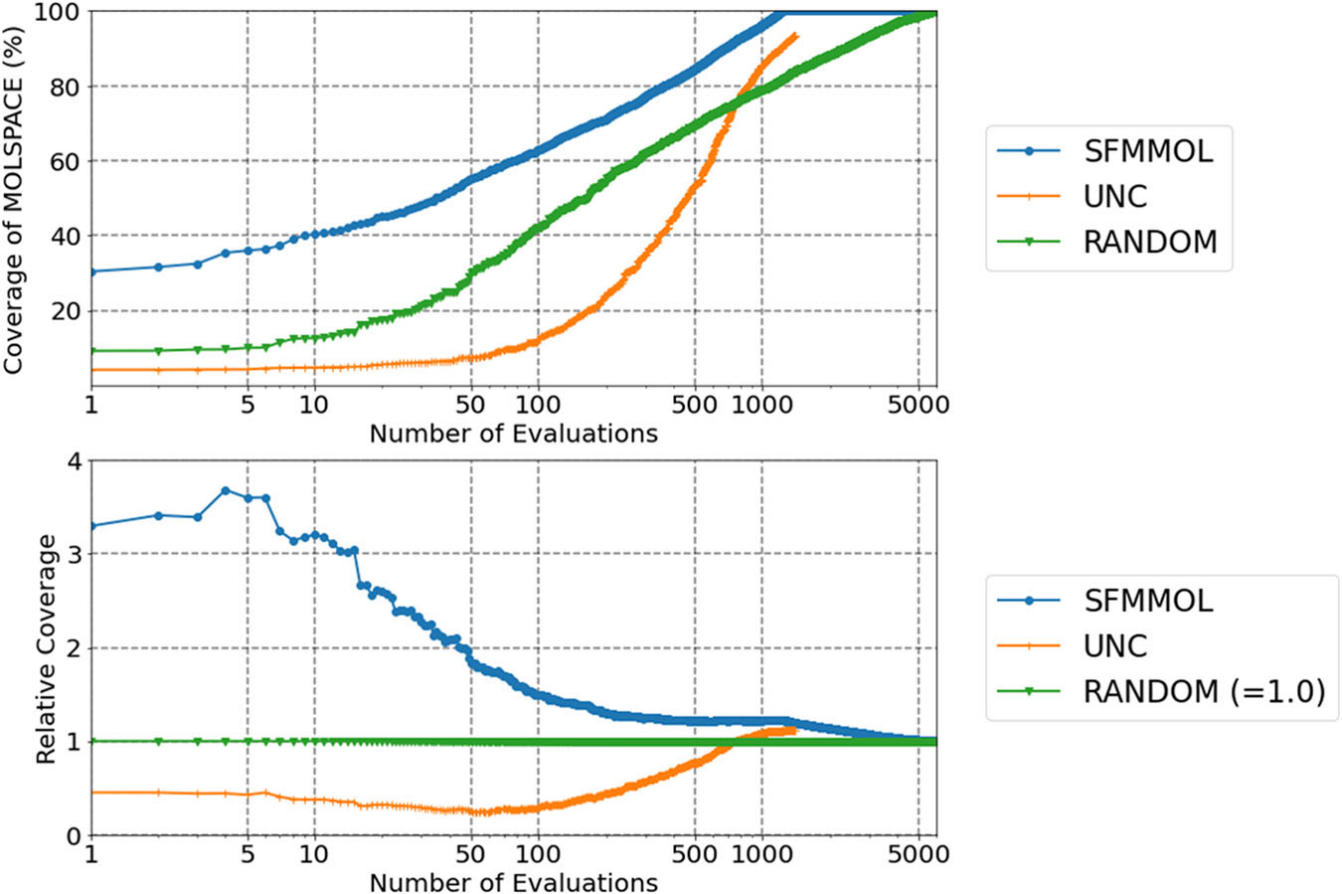
Accumulated coverages of MOLSPACE. By using SFMMOL, uncertainty sampling (UNC), and random sampling (RANDOM), accumulated coverages of MOLSPACE are displayed as functions of number of evaluations (molecules). *Y* axis of bottom figure represents values relative to those of RANDOM. UNC calculation was terminated at 1400 evaluations due to a technical issue: exceeding the limit of memory usage (>16 Giga bytes). SFMMOL remarkably improved coverages, especially in smaller number of evaluations (<100), whilst UNC drastically increased as number of evaluations approached 500.

Initially, the molecules in ACCESSIBLE were evaluated and those in GENERATED were prioritized for evaluation in the subsequent step. To prioritize the molecules, we presumed that structurally similar molecules yield similar properties (solvent-solubilities)^69^. Although the selected synthetic processes were relatively simple, the synthesis of a new molecule was challenging and expensive. Thus, to select an appropriate subset securing the coverage of the entire molecular set, we developed the SFM algorithm for molecular search (SFMMOL). The application of SFMMOL on GENERATED produced a priority list of 1000 molecules along with their ‘coverage’ of MOLSPACE (= ACCESSIBLE ∪ GENERATED), as depicted in Fig. 4 (implementation of SFMMOL and definition of ‘coverage’ are described in the Methods section). Compared to simple random sampling (RANDOM) or BO-like uncertainty sampling (UNC), the SFMMOL remarkably improved the coverages, especially for smaller number of evaluations (<100). In particular, the SFMMOL attained a coverage of 36% (40%, 63%) for 5 (10, 100) molecules in the MOLSPACE, whereas that obtained by RANDOM and UNC was ~10% (13%, 42%) and 4% (5%, 12%), respectively. The results of evaluations for other random sampling algorithms are in Supplementary Figs. 15–17. In addition to the 10 TPP derivatives with similar molecules from ACCESSIBLE, five TPP derivatives with the highest priorities obtained from GENERATED are presented in Fig. 2b, c. Consequently, the five molecules from the top-ranked molecules were synthesized and measured in this research (See Supplementary Method for the details of the preparation of the TPP derivatives **11**–**15**).

### Evaluation and analyses of spectra data

**Fig. 5:**
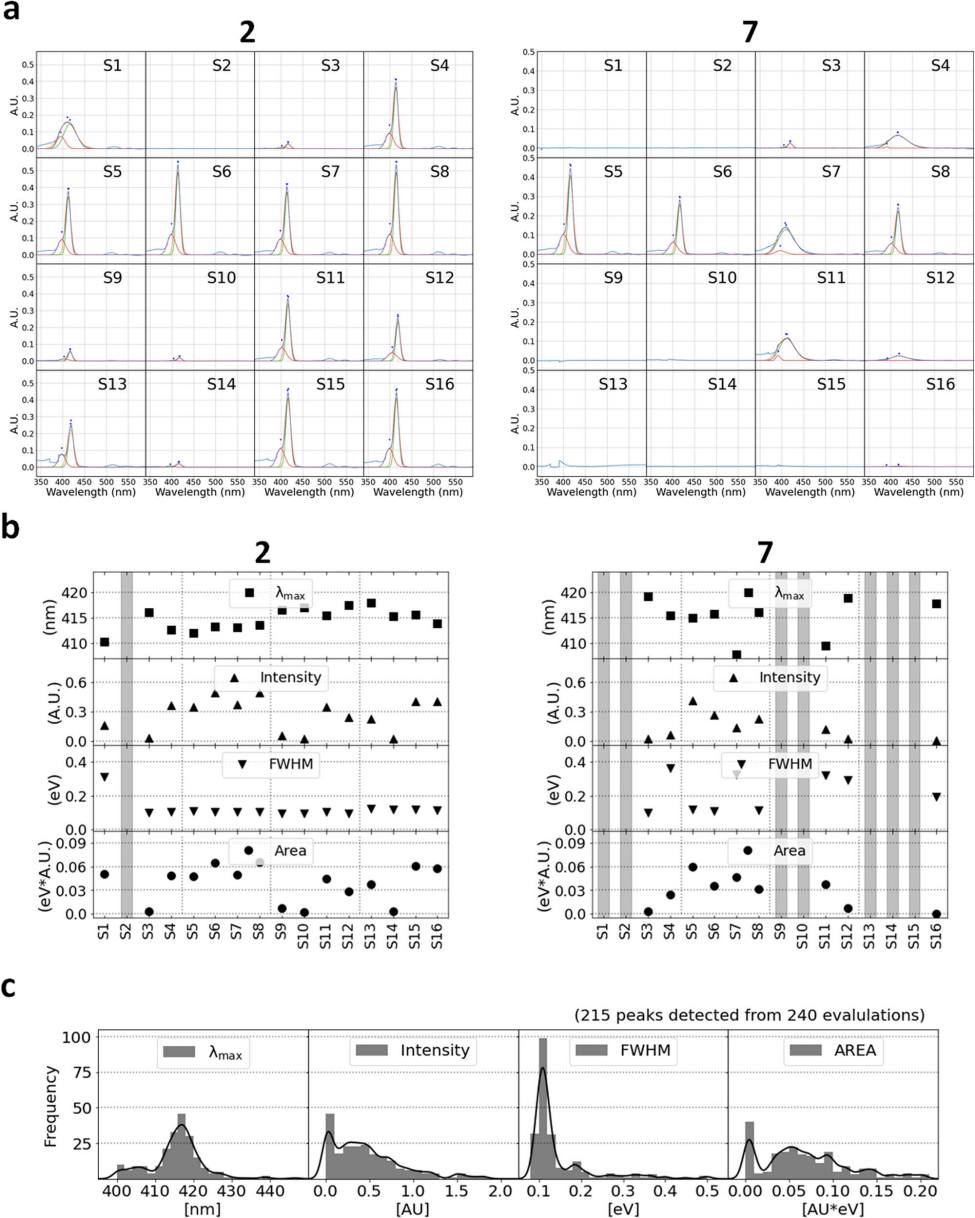
UV–vis absorption spectra of TPP derivatives in 16 solvents. **a** Spectra of TPP derivatives **2** and **7** in 16 solvents. The original spectra were plotted in blue lines. Major peaks around 420 nm were fit using single-Gaussian (orange lines) and two-Gaussian (green and red lines). **b** Four indicator values (${{{{{{\boldsymbol{\lambda }}}}}}}_{{{\max }}}$, Intensity, FWHM, and Area) for **2** and **7** were considered from Gaussian-fittings. Hatched columns represent cases with no peak detected around 420 nm. **c** Histograms of four indicators for all 240 evaluations; main peaks detected for 215 spectra and no main peaks detected for 25 spectra.

In Fig. 5, the spectra of **2** and **7** are depicted in (a), the four indicators of the main peaks (the wavelength of maximum absorbance (λ_max_), the maximum absorbance (Intensity), the full width at half maximum (FWHM), and the area of the peak (Area)) are portrayed in (b), and the histograms of the four indicators for all the major measured and detected peaks are plotted in (c). The process of evaluating the indicators is detailed in the Methods section. As observed, the solute–solvent interaction modified λ_max_, whereas molecular aggregation influenced all four indicators. As the absorption property of the aggregates varied from that of the non-aggregated molecule, the resulting spectra represented the sum of distinct spectra with aggregates. Consequently, the Intensity decreased as the FWHM increased, and the Area was proportional to the number of absorbed photons, which is related to the number of solvated molecules. In principle, no value represents no peak detection, i.e., the molecule is insoluble. More specifically, **2** exhibited a large sharp peak at ~415 nm for 10 solvents (**S4**, **S5**, **S6**, **S7**, **S8**, **S11**, **S12**, **S13**, **S15**, and **S16**) and a small sharp peak for four solvents (**S3**, **S9**, **S10**, and **S14**), whereas **7** displayed a large sharp peak for three solvents (**S5**, **S6**, and **S8**) and a small sharp peak for **S3**. Although no peaks displayed by **2** indicated insolubility for **S2**, **7** was insoluble for seven solvents (**S1**, **S2**, **S9**, **S10**, **S13**, **S14**, and **S15**). Moreover, **2** exhibited a broad peak for only **S1**, thereby indicating its aggregation in water, whereas **7** was aggregated in five solvents (**S4**, **S7**, **S11**, **S12**, and **S16**); λmax: 415 ± 6 nm; the Intensity and Area varied similarly.

**Fig. 6:**
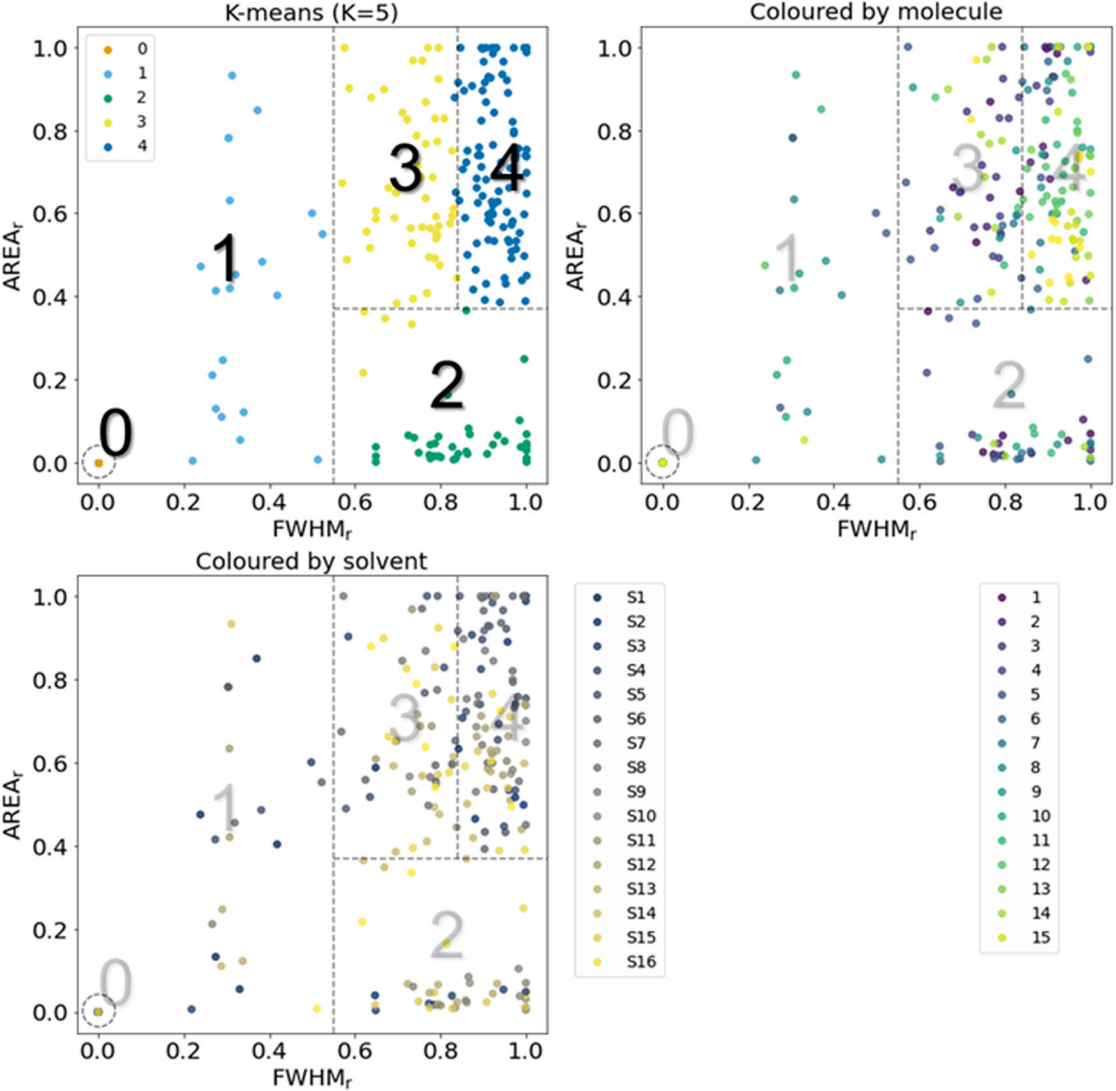
Mapping all 240 evaluations over FWHM_r_ and Area_r_. Colored points in top-left figure follow K-means (*K* = 5) clustering and are simply categorized into five categories (0–4) according to values for each axis based on clusters. Colored points in top-right and bottom figures depict types of molecules in MW order and solvents in epsilon order, respectively.

According to the simulated UV–Vis absorption spectra obtained from the time-dependent density functional theory (TDDFT), representing the solute–solvent interaction but not the influences of the aggregates, the exclusive major peak in the Soret band typically existed for each molecule in any solvent. However, the FWHM varied for each molecule (See Supplementary Fig. 6). To compare the spectra with each other based on these indicators, we used the following two values relative to the most suitable ones for each molecule: FWHM_r_ (= FWHM_min_/FWHM) and Area_r_ (= Area/Area_max_), assuming that each molecule contained at least one appropriate solvent (adequate solubility and/or no aggregates), and the most suitable molecule displayed the sharpest and/or the largest major peak. FWHM_r_ and Area_r_ varied from 0 to 1, and a solvent was deemed as the most suitable if the values were approximate to 1. The mapping of all results (15 molecules × 16 solvents = 240 evaluations) over the FWHM_r_ and Area_r_ are plotted in Fig. 6. In the top-left figure, the points evaluated by K-means clustering (*K* = 5) are denoted with colors for simple categorization according to the values for each axis (each category is surrounded by dashed lines). We simply categorized the molecules from category 0–4, e.g., 0: insoluble (no peak); 1: aggregated (broad peak); 2: less-soluble (small area); 3: slightly aggregated (slightly broader peak); 4: good solvent (sharp and large peak). In the top-right and bottom figures in Fig. 6, the colored points represent the kinds of molecules in MW order or solvents in epsilon order, respectively. As the points of each molecule or solvent spanned a wide area, the governing rules for the indicators were unclear. The category numbers for all evaluations are listed in Supplementary Table 1, wherein the cell color varies from deep to light blue to indicate the classification (0–4). **S1** and **S2** contained several insoluble (0), aggregated (1), and less-soluble (2) molecules, whereas **S5** and **S8** exhibited adequate solubility (3 or 4) for all molecules. Moreover, **7** and **9** comprised several insoluble (0) and aggregated (1) cases, whereas **12** and **14** included several good solvents (4). Although the category number was apparently related to the functional groups (e.g., carboxyl group), multiple irregular behaviors were observed, e.g., **9** is soluble (4) for **S2** but **7** is not (0). Owing to the indistinguishable trend characteristics, we extracted the required information using data-driven approaches in the following subsection.

### Prediction of good solvent and indicators

**Fig. 7:**
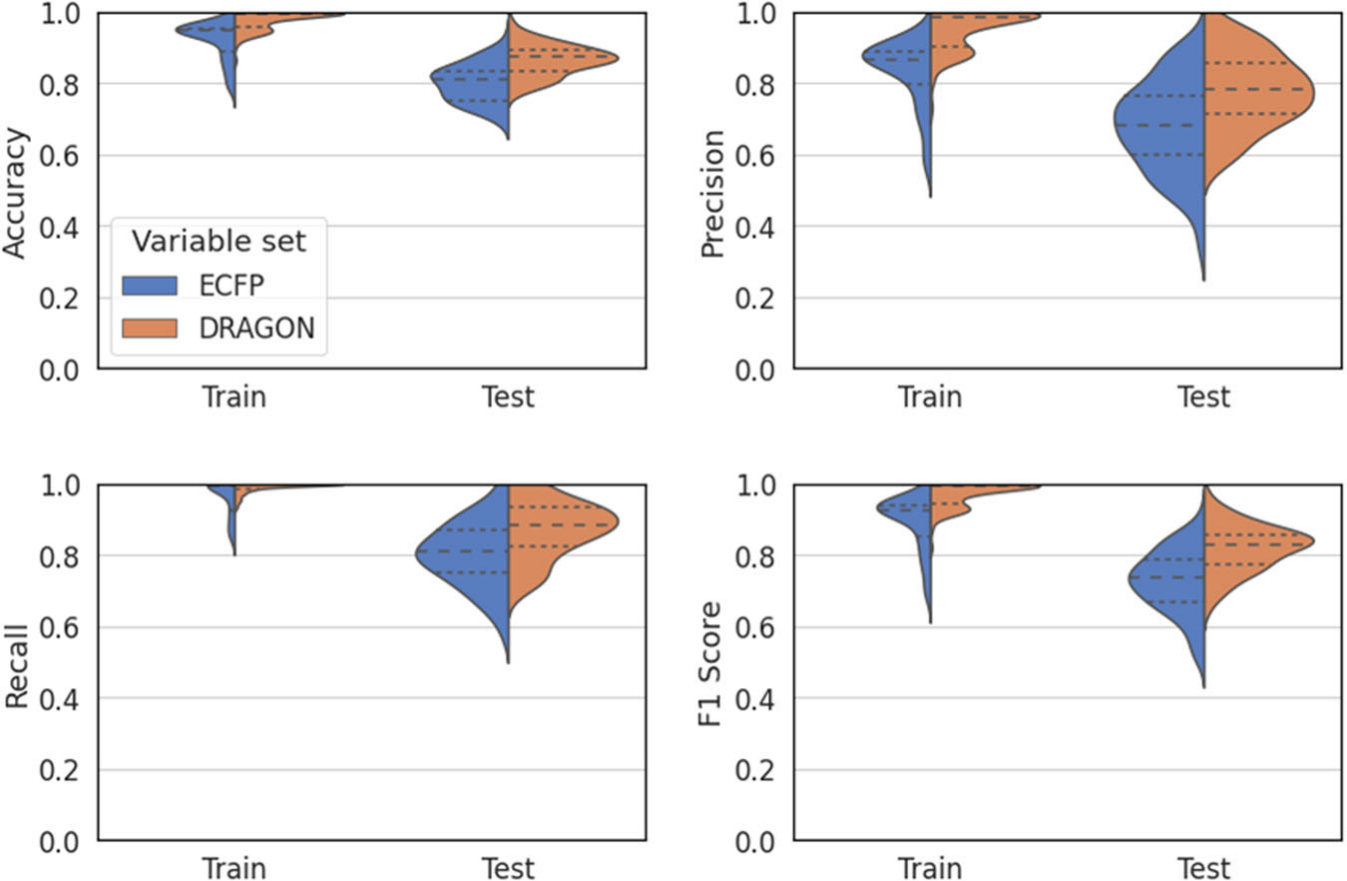
Violin plots for the results of the two-class classification. The distributions of four metrics (accuracy, precision, recall, and f1 score) of the two-class classification for two variable sets (ECFP and DRAGON) are depicted in the violin plots. The dashed lines represent quartiles (25%, 50%, and 75%). The performance of the model with DRAGON outperforms that with ECFP for each metrics. The lower quartiles indicate that the random forests classifier (RFC) generated practical classification models for DRAGON with accuracies >0.83 (precisions >0.71, recalls >0.83, and f1 scores >0.77), whereas slightly inferior models for ECFP with accuracies >0.75 (precisions >0.60, recalls >0.75, and f1 scores >0.67).

The classification and regression models were employed on the two input variable sets: extended connective fingerprints (ECFP) and Dragon7 descriptors (DRAGON), which were evaluated for each molecule–solvent pair. The classification models predicted the adequate solubility for a pair, whereas the regression models predicted the values of FWHM_r_ and Area_r_. In addition, the developed ML models were trained on randomly sampled 80% of the evaluated 240 datapoints (training set) and tested using the remainder 20% data (test set). Specifically, the models were estimated and evaluated for 100 iterations on datasets randomly sampled from 240 datapoints. The classification results are presented in top half of Table 1 and Fig. 7. The lower quartiles in the violin plots indicate that, in most cases, the random forests classifier (RFC) generated practical classification models for DRAGON with accuracies >0.83 (precisions >0.71, recalls >0.83, and f1 scores >0.77), whereas slightly inferior models for ECFP with accuracies >0.75 (precisions >0.60, recalls >0.75, and f1 scores >0.67). Based on the estimated models, we determined several important variables for both ECFP (Supplementary Fig. 8) and DRAGON (Supplementary Table 5). Specifically, the important ECFP bits for molecules such as methyl group (**A1**) and nominal butyl group (**A4**) induced lipophilicity in molecules and rendered them soluble in oily solvents with lipophilic group, e.g., methyl group (**B2**). In contrast, intermediate molecular structures with oxygen (**A2**, **A5**, and **A6**) containing a lone pair either formed hydrogen bonds within the molecules to diminish their solubility in hydrophobic solvents, or formed hydrogen bonds between the molecule and water-like solvent with hydrophilic group (e.g., hydroxy group (**B1**)) to render them soluble. The low-performance tails of the violin plots for ECFP may be caused by that the important bits of the solvents (**B1, B1’**, and **B2**) are too simple to discriminate them, e.g., **S3** (*N*, *N*-dimethylformamide) and **S7** (acetone) have same bit values (see to Supplementary Table 2–4 for details). In addition, the important Dragon descriptors for molecules such as molecular weight related variables (AMW, SLV_MW, and MW) and volumetric variables (Mv, SLV_PDI, and SLV_Vx) contributed interaction within molecules and resulted in molecular aggregation, whereas percentage/number of hydrogen (H% and nH) might result in hydrogen bonding within the molecules or between the molecule and solvent. Lastly, the empirically derived partition coefficient-related variables (P_VSA_LogP_1,7, SLV_MLOGP2, and SLV_ALOGP2) directly influenced the solubility in water-like or oily solvents. Further attempts to predict solubility are depicted in Supplementary Table 8–11.

**Table 1:** Results of two-class classification and regression.

Two-class classification								
Variable set	Accuracy		Precision		Recall		F1 score	
	Train	Test	Train	Test	Train	Test	Train	Test
ECFP	0.92	0.80	0.83	0.68	0.96	0.80	0.89	0.73
SD	0.053	0.054	0.093	0.13	0.051	0.091	0.076	0.088
DRAGON	0.98	0.87	0.95	0.78	0.99	0.87	0.97	0.82
SD	0.027	0.043	0.056	0.10	0.015	0.080	0.035	0.066
Regression								
Variable set	FWHM_r_ *R*^2^		FWHM_r_ MAE		Area_r_ *R*^2^		Area_r_ MAE	
	Train	Test	Train	Test	Train	Test	Train	Test
ECFP	0.90	0.49	0.070	0.14	0.87	0.50	0.090	0.18
SD	0.027	0.21	0.0073	0.022	0.046	0.11	0.015	0.020
DRAGON	0.91	0.59	0.059	0.12	0.93	0.60	0.069	0.17
SD	0.034	0.13	0.011	0.021	0.016	0.080	0.0074	0.017

Top: Two-class classification (appropriate solvent prediction) based on ECFP or DRAGON. Bottom: Regression of FWHM_r_ and Area_r_ from ECFP or DRAGON. The performance metrics for classification (Accuracy, Precision, Recall, and F1 score) and those for regression (coefficient of determination *R*^2^ and mean absolute error (MAE)) are calculated by Scikit-learn^76^. The first value in each cell displays the mean, and the second value displays the standard deviation (SD).

The regression results obtained after 100 iterations of evaluation for the randomly sampled datasets are listed in the bottom half of Table 1, which implied that the random forests regression (RFR) reasonably predicted the FWHM_r_ and Area_r_ based on the molecular structures described by ECFP and DRAGON. However, the performances were not optimal, and the means of coefficient of determination *R*^2^ scores ranged within 0.5–0.6. Additional attempts to estimate the regressors are depicted in Supplementary Fig. 10 and Supplementary Table 12.

## Discussion

The derivation of the optimal evaluation order and the development of accurate prediction models to estimate the solubility of TPP derivatives demonstrated the ability of semi-automated experiment schemes for material exploration. The current research was conducted following three technological advancements. First, we developed a practical scheme to set the targeted chemical space of TPP derivatives based on accessible materials. In particular, the chemical space (MOLSPACE) generated from three simple one-step reactions appropriately covered most of the space defined by existing molecular structures (source: PubChem). Second, we employed a robotic experiment system integrated with UV–Vis absorption spectroscopy and analysis python script to generate the truly FAIR dataset with failure data (insoluble cases) for the solubility of TPP derivatives. Ultimately, we formulated and implemented the efficient molecular selection problem as SFMMOL, wherein the molecules could be selected from 1.5–3.5 or 5–9 times larger coverage of MOLSPACE compared to that of the random sampling or the BO-like uncertainty sampling, respectively, as well as exhibiting comparable prediction performance; mean absolute error of the prediction models obtained by SFMMOL is 0.82–1.2 or 0.66–0.99 times that of the random sampling or the uncertainty sampling (refer to Supplementary Note 2 and Supplementary Figs. 12–14). Evidently, the penalty term introduced to avoid duplication in SFMMOL effectively improved the prediction performances of the ML models (refer to Supplementary Note 2 and Supplementary Figs. 12–14). Moreover, the subfunctions and parameter settings of SFMMOL can be extended, and thus, further research is required to discover the optimal settings for various targeted properties, e.g., utilization of estimation challenges in property prediction, considering molecular accessibility in terms of commercial availability or ease of synthesis such as RouteScore^70^. Despite the limited number of evaluated molecules, the above-mentioned advancements enabled the development of qualitative prediction models that could accurately predict the solubility of TPP derivatives securing the coverage of the targeted chemical space.

Compared to similar approaches such as high-throughput experimentation or design of experiments, the current scheme is advantageous for application in the early stages of material search. The proposed scheme offers a rational and efficient order of evaluations without considering pre-existing data or selection of variables, whereas the existing schemes, e.g., random sampling or a BO-like algorithm, selected a small number of variables for exploration; otherwise, the material search would require extensive evaluations. Furthermore, the proposed scheme facilitated the exploration of real materials owing to integrated functionalities of synthesis, measurements, and analyses.

Nonetheless, we acknowledge that the proposed scheme poses certain limitations. First, the synthetic processes are limited and not entirely automated. In addition, multistep synthetic processes including other couplings such as metal substitution should be considered, and the process parameters should be automatically optimized. Second, the proposed method delivered adequate performance in the early stage of material search, but in future, the searching algorithm would be extended by combining with existing algorithms such as BOs. Moreover, the indicators FWHM and Area are regarded as approximate shape parameters of the UV–Vis absorption spectrum, and the prediction of the spectrum shape is still a challenging task^71^; thus, in future, we will continue to improve the prediction performance of the regression models. Despite these limitations, the proposed scheme demonstrated an effective example for material search projects.

To analyze the material properties based on UV–Vis absorption spectra, the four indicators provided sufficient information regarding the solubility of the TPP derivatives. Moreover, the definition of relevant indicators for the spectra can enable the same automated experiment scheme to characterize more complicated properties related to the UV–Vis absorption spectra of materials, such as vibronic coupling and degree of aggregates. Furthermore, the connection of another measurement instrument such as fluorometer and particle-size analyzer can extend the application of this system over a wider range of material search projects.

## Methods

### Submodular function maximization for molecular search

To select the molecules that effectively cover the targeted chemical space, we regarded the problem as an SFM to maximize the coverage for the space, where $V$ denotes a finite set, a set function $f:{2}^{V}{\mathbb{\to }}{\mathbb{R}}$ is *submodular*, if for every $A\subseteq B\subseteq V$ and $e\in V\setminus B$, it holds the following condition^55^:

1 $$\begin{array}{c}f\,\left(A\cup \left\{e\right\}\right)\,-\,f\,\left(A\right)\,\ge \,f\,\left(\,B\,\cup \,\left\{e\right\}\right)\,-\,f\,\left(B\right).\end{array}$$

The target priority function of the current problem is defined as a cover function for molecular sets $S,{T}\subseteq {{{{{\rm{MOLSPACE}}}}}}$ as follows:

2 $${f}_{{{{{{{{\rm{cov}}}}}}}}}\left(S\right)\,=\,\mathop{\sum}\limits_{{m}_{j}\,\in \,{\cup{_{m_{i}\,\in \,S}}{C}_{{{{{{{{\rm{MOLSPACE}}}}}}}}}\left({m}_{i}\right)}}w\left({m}_{j}\right),$$

3 $${C}_{T} \left({m}_{i}\right) \, = \, \left\{{m}_{j} \in T: {m}_{j}\, {{{{{\rm{is}}}}}}\; {{{{{\rm{covered}}}}}}\; {{{{{\rm{by}}}}}}\,{m}_{i}\right\},$$

where $w\left({m}_{j}\right)$ returns weight for a covered molecule ${m}_{j}$, ${C}_{T}\left({m}_{i}\right)$ returns a molecular set ($\subseteq T$) covered by a selected molecule ${m}_{i}$. For every molecular selection $S\subseteq T\subseteq {{{{{\rm{MOLSPACE}}}}}}$ and $m\in {{{{{\rm{MOLSPACE}}}}}}\setminus T$, ${f}_{{{{{{\rm{cov}}}}}}}$ holds that

4 $$\begin{array}{c}{f}_{{{{{{\rm{cov}}}}}}}\left(S\,\cup \,\left\{m\right\}\right)\,-\,{f}_{{{{{{\rm{cov}}}}}}}\left(S\right)\,\ge\, {f}_{{{{{{\rm{cov}}}}}}}\left(T\,\cup \,\left\{m\right\}\right)-{f}_{{{{{{\rm{cov}}}}}}}\left(T\right),\end{array}$$

because the influence of the newly selected molecule ${m}_{i}$ for covering the new molecules apparently decreased as the total selected molecules increased. Therefore, ${f}_{{{{{{\rm{cov}}}}}}}$ is submodular. Subsequently, we implemented the SFMMOL based on the greedy algorithm that can efficiently solve this problem^55^. The general form of the priority function for the $i$-th molecular selection can be defined as follows:

5 $${f}_{{{{{{\rm{SFMMOL}}}}}}}^{i}\left(\left\{{m}_{i}\right\}\right)\,=	\, \left({f}_{{{{{{\rm{cov}}}}}}}\,\left({M}_{{{{{{\rm{EVALUATED}}}}}}}^{\left[1:\,i-1\right]}\,\cup \left\{{m}_{i}\right\}\right)\,-\,{f}_{{{{{{\rm{cov}}}}}}}\,\left({M}_{{{{{{\rm{EVALUATED}}}}}}}^{\left[1:\,i-1\right]}\right)\right)\\ 	-\lambda * {W}_{{{{{{\rm{tot}}}}}}}* \left({g}_{{{{{{\rm{pen}}}}}}}\,\left({M}_{{{{{{\rm{EVALUATED}}}}}}}^{\left[1:\,i-1\right]}\,\cup \left\{{m}_{i}\right\}\right)\right.\\ 	\left.\,-\,{g}_{{{{{{\rm{pen}}}}}}}\,\left({M}_{{{{{{\rm{EVALUATED}}}}}}}^{\left[1:\,i-1\right]}\right)\right),$$

where ${M}_{{{{{{\rm{EVALUATED}}}}}}}^{\left[1:{i}-1\right]}$ ($=\left\{{m}_{1},\ldots ,{m}_{i-1}\,\right\}$) represents an evaluated (selected) molecular set, $\lambda$ (= 0–1) denotes a parameter balancing the two terms, ${W}_{{{{{{\rm{tot}}}}}}}$ denotes the total weight of MOLSPACE, and ${g}_{{{{{{\rm{pen}}}}}}}$ indicates a penalty function for covered molecules. The first term maximizes the cover function for newly covered molecules, whereas the second term minimizes the penalty for newly covered molecules. If $\lambda =0$ (simplest case), ${f}_{{{{{{\rm{SFMMOL}}}}}}}^{i}\,$ is maximized by selecting a molecule that attained the largest value in the first term, and the penalty term was neglected. ${W}_{{{{{{\rm{tot}}}}}}}$ and ${f}_{{{{{{\rm{pen}}}}}}}$ were defined as follows; for $S\subseteq {{{{{\rm{MOLSPACE}}}}}}$,

6 $${W}_{{{{{{\rm{tot}}}}}}}=\mathop{\sum}\limits_{{m}_{i}\,\in {{{{{\rm{MOLSPACE}}}}}}}w\left({m}_{j}\right),$$

7 $${g}_{{{{{{\rm{pen}}}}}}}\,\left(S\right)=\mathop{\sum}\limits_{{m}_{j}\,\in \,{\cup{_{m_{i}\,\in \,S}}{C}_{{{{{{{{\rm{MOLSPACE}}}}}}}}}\left({m}_{i}\right)}}p\left({m}_{j}\right),$$

where $p\left({m}_{j}\right)$ denotes the penalty weight for a covered molecule ${m}_{j}$. Thus, the expression (5) can be transformed as follows:

8 $${f}_{{{{{{\rm{SFMMOL}}}}}}}^{i}\left(\left\{{m}_{i}\right\}\right)\,=	\, \mathop{\sum}\limits_{{m}_{j}\,\in \,{C}_{{M}_{{{{{{\rm{NOT}}}}}}\_{{{{{\rm{COVERED}}}}}}}^{\left[1:i-1\right]}}\left({m}_{i}\right)}w\left({m}_{j}\right)\,-\lambda * {W}_{{{{{{\rm{tot}}}}}}}\\ 	* \left(\mathop{\sum}\limits_{{m}_{j}\,\in \,{C}_{{M}_{{{{{{\rm{NOT}}}}}}\_{{{{{\rm{COVERED}}}}}}}^{\left[1:i-1\right]}}\left({m}_{i}\right)}p\left({m}_{j}\right)\right),$$

where ${M}_{{{{{{\rm{NOT}}}}}}\_{{{{{\rm{COVERED}}}}}}}^{\left[1:{i}-1\right]}$ (${M}_{{{{{{\rm{COVERED}}}}}}}^{\left[1:{i}-1\right]}$) is not covered (covered) molecular set (${M}_{{{{{{\rm{COVERED}}}}}}}^{\left[1:{i}-1\right]}+{M}_{{{{{{\rm{NOT}}}}}}\_{{{{{\rm{COVERED}}}}}}}^{\left[1:{i}-1\right]}={{{{{\rm{MOLSPACE}}}}}}$). This expression presents a general form of ${f}_{{{{{{\rm{SFMMOL}}}}}}}^{i}$ with three subfunctions and a parameter to be specified: ${C}_{S}\left({m}_{i}\right)$, $w\left({m}_{i}\right)$, $p\left({m}_{i}\right)$, and $\lambda$. For comparison, we implemented two conventional selection algorithms: random sampling (RANDOM) and BO-like uncertainty sampling (UNC). The priority functions of RANDOM and UNC can be described as follows: for $\forall {m}_{i}\,\in\,{{{{{\rm{MOLSPACE}}}}}}\setminus {M}_{{{{{{\rm{EVALUATED}}}}}}}^{\left[1:{i}-1\right]}$,

9 $${f}_{{{{{{\rm{RANDOM}}}}}}}^{i}\left(\left\{{m}_{i}\right\}\right)={URandom}\left(0,\,1.0\right),$$

10 $${f}_{{{{{{\rm{UNC}}}}}}}^{i}\left(\left\{{m}_{i}\right\}\right)={{Uncertainty}}_{{M}_{{{{{{\rm{EVALUATED}}}}}}}^{\left[1:i-1\right]}}\left({m}_{i}\right),$$

where ${URandom}\left(0,1.0\right)$ returns a uniform random number from 0 to 1.0, and ${{Uncertainty}}_{{M}_{{{{{{\rm{EVALUATED}}}}}}}^{\left[1:{i}-1\right]}}\left({m}_{i}\right)$ returns a prediction variance for a molecule ${m}_{i}$ calculated from the Gaussian process regression (a Bayesian method) model estimated for the evaluated molecular set ${M}_{{{{{{\rm{EVALUATED}}}}}}}^{\left[1:{i}-1\right]}$ (least confident selection)^72^. The performances of the algorithms were comparatively analyses based on the ‘coverage’ calculated for the 1st and 2nd neighbors (neighbors of neighbor molecules).

Specifically, in this research, we used the following subfunctions and parameter setting for ${f}_{{{{{{\rm{SFMMOL}}}}}}}^{i}$; for $\forall \,{m}_{i}\in {{{{{\rm{MOLSPACE}}}}}}$ and $S\subseteq {{{{{\rm{MOLSPACE}}}}}}$,

11 $${C}_{S}\left({m}_{i}\right)=\left\{{m}_{j}\,\in \,S\,:\,{d}_{{ij}} \, < \,{D}_{{{{{{\rm{TH}}}}}}}\right\},$$

12 $$w\left({m}_{i}\right)=1,$$

13 $$p({m}_{i})=\left\{\begin{array}{cc}1 & {{{{{\rm{if}}}}}}\,{m}_{i}\in {M}_{{{{{{\rm{EVALUATED}}}}}}}^{[1:i-1]}\\ 0 & {{{{{\rm{else}}}}}}\hfill\end{array}\right.,$$

14 $$\lambda =1,$$

where ${d}_{{ij}}$ represents the Tanimoto distance over ECFP6 between the two molecules (${m}_{i},{m}_{j}$), ${D}_{{{{{{\rm{TH}}}}}}}$ denotes a distance threshold to classify them as neighbors. In this study, the value of ${D}_{{TH}}$ was selected as 0.3 (refer to Supplementary Note 1 and Supplementary Fig. 11). A molecule ${m}_{j}$ is considered ‘covered’ if ${m}_{j}$ or one of its neighbor molecules has been already selected. The expression (12) represents that we equivocally handled all molecules, each with a weight of 1. Therefore, ${W}_{{{{{{\rm{tot}}}}}}}$ equals to the total number of ${{{{{\rm{MOLSPACE}}}}}}$ (${NMOL}$). The expression (13) penalizes the evaluated molecules to be covered and each weight is designated as 1. The parameter setting $\lambda =1$ (14) denotes that we maximized the effect of the penalty to avoid any coverage duplication of the molecules evaluated in the early stage, as it is one of the best parameter settings to secure both the coverage of MOLSPACE and prediction performance (refer to Supplementary Figs. 12–14). Therefore, based on the above subfunctions and parameter setting (11–14), we specified ${f}_{{{{{{\rm{SFMMOL}}}}}}}$ as follows: for $\forall {m}_{i}\in {{{{{\rm{MOLSPACE}}}}}}\setminus {M}_{{{{{{\rm{EVALUATED}}}}}}}^{\left[1:{i}-1\right]}$,

15 $${f}_{{{{{{\rm{SFMMOL}}}}}}}^{i}\left({m}_{i}\right)=	 \,{Count}\left(\left\{{m}_{j}\,\in \,{M}_{{{{{{\rm{NOT}}}}}}\_{{{{{\rm{COVERED}}}}}}}^{\left[1:\,i-1\right]}\,:\,{d}_{{ij}}\, < \,{D}_{{{{{{\rm{TH}}}}}}}\right\}\right)\\ 	-{NMOL}* {Count}\left(\left\{{m}_{j}\,\in \,{M}_{{{{{{\rm{EVALUATED}}}}}}}^{\left[1:\,i-1\right]}\,:\,{d}_{{ij}}\, < \,{D}_{{{{{{\rm{TH}}}}}}}\right\}\right),$$

where ${Count}$ determines the number of items in the given set. The subfunctions and parameter settings for SFMMOL could vary, e.g., defining another distance instead of Tanimoto distance over ECFP, setting a smaller $\lambda$, penalizing covered molecules (${M}_{{{{{{\rm{COVERED}}}}}}}^{\left[1:{i}-1\right]}$) or costly molecules instead of ${M}_{{{{{{\rm{EVALUATED}}}}}}}$, using certain probability distributions for $w\left({m}_{i}\right)$ or $p\left({m}_{i}\right)\,$ instead of deterministic functions. Note that the appropriate subfunctions and parameter settings depend on the targeted property of the molecular search.

### Automated experiment

**Fig. 8:**
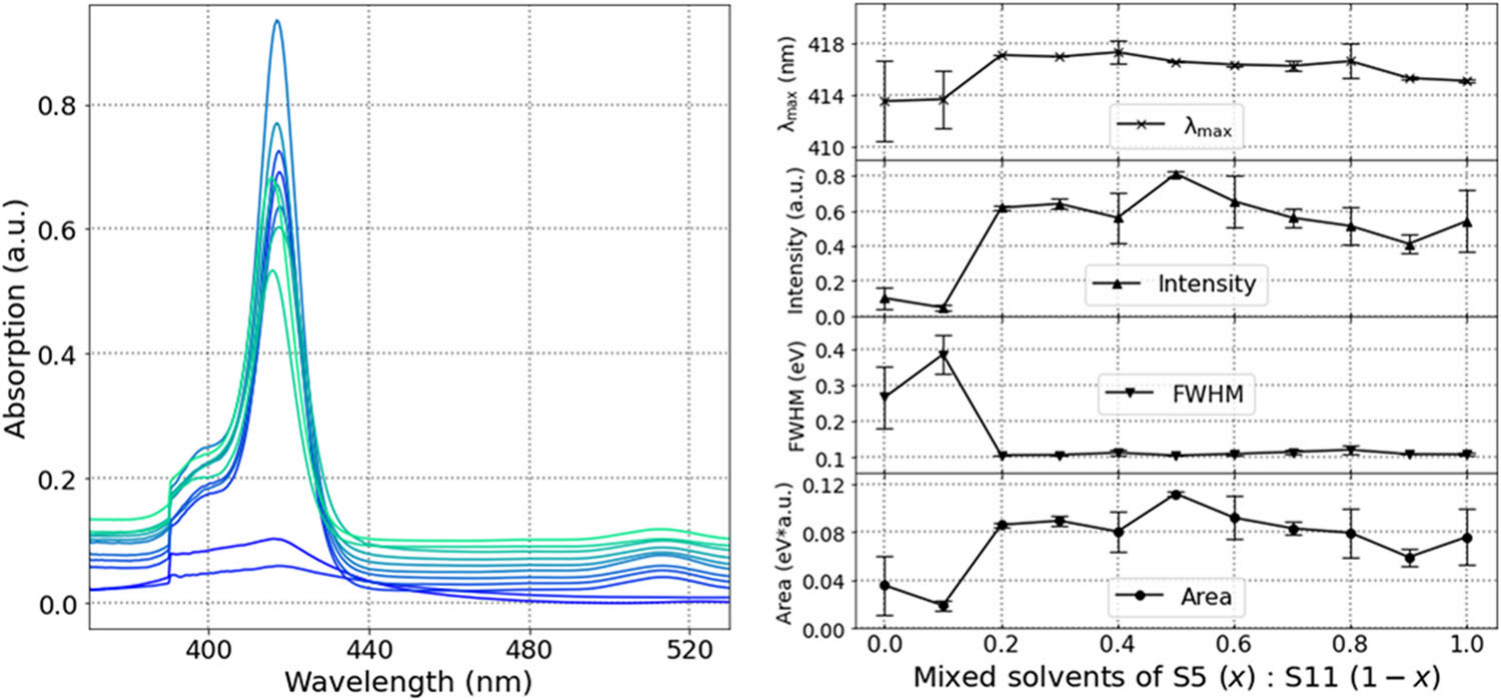
UV–Vis absorption spectra of 7 in mixed solvents of S5 (methanol) and S11 (tetrahydrofuran). **a** change of the spectra of 7 along with the mixing ratio of solvents **b** spectra characteristics (*λ*_max_, Intensity, FWHM, and Area) considered from major peaks using the method explained in the following segment.

We utilized the automated equipment with UV–Vis spectroscopy to obtain solubility data of TPPs of which solubilities were unknown. To measure the UV–Vis absorption spectra, the TPPs were initially diluted with tetrahydrofuran to 1.0 × 10^–4^ mol/L. Thereafter, the solution of 100 μL was diluted to 2.0 × 10^–6^ mol/L with solvents of 4900 μL, respectively. The solutions were injected to the UV–Vis absorption spectrometer with a flow cell (optical path length = 10 mm), and the spectra were measured. In addition, the baselines were also measured using a mixed solvent (THF/solvent: 100 μL/4900 μL) to subtract from the spectra of the solutions of TPPs. The sample measurements along with the four indicators for **7** in mixed solvents of soluble methanol and less-soluble THF with varying mixture ratios are depicted (Fig. 8a). Intensity and Area increased gradually along with the quantity of methanol, whereas FWHM decreased to ~0.1 eV for *x* = 0.3, and it remained low thereafter (Fig. 8b).

### Spectrum analysis

The solubilities of the molecules were compared based on the UV–Vis absorption spectra focusing on the major peaks at ~400 nm. Instead of using completely raw values from the spectrum, we selected four characteristics, e.g., peak position (*λ*_max_), peak height (Intensity), peak width (FWHM), and peak area (Area) (Supplementary Fig. 6). Based on the TDDFT calculations (Supplementary Fig. 7), every selected molecule would exhibit this sharp Soret band. Although a common peak existed at ~520 nm (Q band) for the molecules, it was much smaller and unsuitable for comparison. The peak characteristics could differ with the environmental effects on the molecules: molecule concentration, structures of solvation or aggregation of themselves, temperature, and atmospheric pressure. The automated experiment eliminated the variations from the last two environmental factors for all the measurements. According to the TDDFT calculations (Supplementary Fig. 7) for various solvents, the estimated influence on the spectra was limited to the shift of *λ*_max_. As dimerization (simplest molecular aggregation) produces shifted-ESs in comparison to those of the single molecule, the primary peak broadened (wider FWHM) owing to the existence of several shifted ESs^73,74^. Thus, we used the FWHM as an indicator of solubility/aggregation of the dye molecules. Moreover, the Area was proportional to the total number of absorbed photons, which is proportional to the number of dye molecules in the solution. This method was used to measure the solubility of poor-soluble cases.

To select the characteristics (*λ*_max_, Intensity, FWHM, and Area) of the main peak, we used single-Gaussian-fitting for the first three values and two-Gaussian-fitting for the remaining value. In particular, the two Gaussian-fitting was applied for Area, because the existence of the shorter-wavelength side at the shoulder of the main peak considerably influenced the value.

### Construction of ML models

The ML models were developed to predict the peak-characteristic values (regression) and assess the solubility of the solute–solvent combination (classification). The schematics are presented in Supplementary Fig. 9. Accordingly, the models were developed using the random forest regression and the classifier provided by Scikit-learn, which are popular for predicting material properties^75,76^. In addition, we used the popular ECFP and Dragon descriptors to compare the input variables^77^. Specifically, 80% of the entire data was used as the training dataset, and the remaining 20% was utilized as the test dataset. The performances of the regression models were measured as the mean absolute error on the test dataset, whereas those of the classifier was measured based on accuracy. More specifically, two hyperparameters (n_estimators and max_depth) were optimized by grid search with fivefold cross-validation; n_estimators was optimized from {100, 300, 500}, and max_depth was optimized from {2, 3, …, 10}. To determine the important variables, we used a Boruta algorithm that internally employed random forests for the significance tests of variables^78^.

## Supplementary information

Supplementary Information

Description of Additional Supplementary Files

Supplementary Data 1

## Data Availability

All data generated during this research are provided in Supplementary Note 1, 2 and Supplementary Data 1 (10.6084/m9.figshare.21308337).

## Code availability

The custom code used in this study has been made publicly available on GitHub at https://github.com/rakushirasawa/SfmMol under the MIT open source license.

## References

[1] Coley CW, Eyke NS, Jensen KF (2020). Autonomous discovery in the chemical sciences part I: progress. Angew. Chem. Int. Ed..

[2] Coley CW, Eyke NS, Jensen KF (2020). Autonomous discovery in the chemical sciences part II: outlook. Angew. Chem. Int. Ed..

[3] Häse F, Roch LM, Aspuru-Guzik A (2019). Next-generation experimentation with self-driving laboratories. Trends Chem..

[4] Stein HS, Gregoire JM (2019). Progress and prospects for accelerating materials science with automated and autonomous workflows. Chem. Sci..

[5] Breen CP, Nambiar AMK, Jamison TF, Jensen KF (2021). Ready, set, flow! Automated continuous synthesis and optimization. Trends Chem..

[6] Desai B (2013). Rapid discovery of a novel series of Abl kinase inhibitors by application of an integrated microfluidic synthesis and screening platform. J. Med. Chem..

[7] Weber L, Wallbaum S, Broger C, Gubernator K (1995). Optimization of the biological activity of combinatorial compound libraries by a genetic algorithm. Angew. Chem. Int. Ed. Engl..

[8] Porwol L (2020). An autonomous chemical robot discovers the rules of inorganic coordination chemistry without prior knowledge. Angew. Chem. Int. Ed..

[9] Nikolaev, P. et al. Autonomy in materials research: a case study in carbon nanotube growth, *NPJ Comput. Mater*. **2**, 16031 (2016).

[10] Tabor DP (2018). Accelerating the discovery of materials for clean energy in the era of smart automation. Nat. Rev. Mater..

[11] Zheng Q (2019). Anisotropic polyoxometalate cages assembled via layers of heteroanion templates. J. Am. Chem. Soc..

[12] Sans V, Cronin L (2016). Towards dial-a-molecule by integrating continuous flow, analytics and self-optimisation. Chem. Soc. Rev..

[13] Sans V, Porwol L, Dragone V, Cronin L (2015). A self-optimizing synthetic organic reactor system using real-time in-line NMR spectroscopy. Chem. Sci..

[14] Christensen M (2021). Data-science driven autonomous process optimization. Commun. Chem..

[15] Jung HS (2018). Organic molecule-based photothermal agents: an expanding photothermal therapy universe. Chem. Soc. Rev..

[16] Montaseri H, Kruger CA, Abrahamse H (2020). Recent advances in porphyrin-based inorganic nanoparticles for cancer treatment. Int. J. Mol. Sci..

[17] Zou Q (2017). Biological photothermal nanodots based on self-assembly of peptide-porphyrin conjugates for antitumor therapy. J. Am. Chem. Soc..

[18] Li LL, Diau EWG (2013). Porphyrin-sensitized solar cells. Chem. Soc. Rev..

[19] Imahori H, Umeyama T, Ito S (2009). Large π-aromatic molecules as potential sensitizers for highly efficient dye-sensitized solar cells. Acc. Chem. Res..

[20] Lee MW, Lee DL, Yen WN, Yeh CY (2009). Synthesis, optical and photovoltaic properties of porphyrin dyes. J. Macromol. Sci. Part A Pure Appl. Chem..

[21] Monobe H, Mima S, Sugino T, Shimizu Y (2001). Mesomorphic and photoconductive properties of a mesogenic long-chain tetraphenylporphyrin Nickel (II) complex. J. Mater. Chem..

[22] Borders B (2017). Photoconductive behavior of binary porphyrin crystalline assemblies. J. Porphyr. Phthalocyanines.

[23] Shimizu Y, Tomonorifuchita, Higashiyama T, Sugino T (1999). Photocurrent action spectra of the photoconductive cell with a mesogenic long-chain tetraphenylporphyrin. Mol. Cryst. Liq. Cryst. Sci. Technol. Sect. A. Mol. Cryst. Liq. Cryst..

[24] Anderson HL (1994). Conjugated porphyrin ladders. Inorg. Chem..

[25] Yokoyama T, Yokoyama S, Kamikado T, Okuno Y, Mashiko S (2001). Selective assembly on a surface of supramolecular aggregates with controlled size and shape. Nature.

[26] Sasaki N (2020). Supramolecular double-stranded Archimedean spirals and concentric toroids. Nat. Commun..

[27] Kobayashi, T. *J-Aggregates*, Vol. 2 (World Scientific, 2012).

[28] Gustavo G. et al. Effects of meso-tetrakis (4-sulfonatophenyl) porphyrin (TPPS4) aggregation on its spectral and kinetic characteristics and singlet oxygen production, *Spectrochimica Acta Part A: Molecular and Biomolecular Spectroscopy* **261**, 120063 (2021) 10.1016/j.saa.2021.120063 34153547

[29] Hasobe T (2012). Photo- and electro-functional self-assembled architectures of porphyrins. Phys. Chem. Chem. Phys..

[30] Hu X (1998). Architecture and mechanism of the light-harvesting apparatus of purple bacteria. Proc. Natl Acad. Sci. USA.

[31] Gust D, Moore TA, Moore AN (1993). Molecular mimicry of photosynthetic energy and electron transfer. Acc. Chem. Res..

[32] McDermott G (1995). Crystal structure of an integral membrane light-harvesting complex from photosynthetic bacteria. Nature.

[33] Wasielewski MR (2009). Self-assembly strategies for integrating light harvesting and charge separation in artificial photosynthetic systems. Acc. Chem. Res..

[34] Kim S (2021). PubChem in 2021: New data content and improved web interfaces. Nucleic Acids Res..

[35] Rogers D, Hahn M (2010). Extended-connectivity fingerprints. J. Chem. Inf. Model..

[36] Bajusz D, Rácz A, Héberger K (2015). Why is Tanimoto index an appropriate choice for fingerprint-based similarity calculations?. J. Cheminform..

[37] Cortés-Borda D (2018). An autonomous self-optimizing flow reactor for the synthesis of natural product carpanone. J. Org. Chem..

[38] Houben C, Lapkin AA (2015). Automatic discovery and optimization of chemical processes. Curr. Opin. Chem. Eng..

[39] Kiyohara S, Miyata T, Tsuda K, Mizoguchi T (2018). Data-driven approach for the prediction and interpretation of core-electron loss spectroscopy. Sci. Rep..

[40] Nemykin VN, Hadt RG (2010). Interpretation of the UV−Vis spectra of the meso(ferrocenyl)-containing porphyrins using a TDDFT approach: is gouterman’s classic four-orbital model still in play?. J. Phys. Chem. A.

[41] Ehrenreich P (2016). H-Aggregate analysis of P3HT thin films-capability and limitation of photoluminescence and UV/Vis spectroscopy. Sci. Rep..

[42] Ouyang C, Chen S, Che B, Xue G (2007). Aggregation of azo dye orange I induced by polyethylene glycol in aqueous solution. Colloids Surf., A Physicochem. Eng. Asp..

[43] Neumann B, Huber K, Pollmann P (2000). A comparative experimental study of the aggregation of acid red 266 in aqueous solution by use of 19F-NMR{,} UV/Vis spectroscopy and static light scattering. Phys. Chem. Chem. Phys..

[44] Würthner F, Kaiser TE, Saha-Möller CR (2011). J-Aggregates: from serendipitous discovery to supramolecular engineering of functional dye materials. Angew. Chem. Int. Ed..

[45] Bodor N, Gabanyi Z, Wong CK (1989). A new method for the estimation of partition coefficient. J. Am. Chem. Soc..

[46] Hansen, C. M. The three dimensional solubility parameter. Danish Tech. 14 (Copenhagen, 1967).

[47] Stefanis E, Panayiotou C (2012). A new expanded solubility parameter approach. Int. J. Pharm..

[48] Boobier S (2020). Machine learning with physicochemical relationships: solubility prediction in organic solvents and water. Nat. Commun..

[49] Ye Z, Ouyang D (2021). Prediction of small-molecule compound solubility in organic solvents by machine learning algorithms. J. Cheminform..

[50] Abraham MH (2010). Prediction of solubility of drugs and other compounds in organic solvents. J. Pharm. Sci..

[51] Rodrigues T (2019). The good, the bad, and the ugly in chemical and biological data for machine learning. Drug Discov. Today Technol..

[52] Callaghan S (2021). Toward machine learning-enhanced high-throughput experimentation for chemistry. Patterns.

[53] Taniguchi M, Lindsey JS, Bocian DF, Holten D (2021). Comprehensive review of photophysical parameters (ε, Φf, Τs) of tetraphenylporphyrin (H2TPP) and zinc tetraphenylporphyrin (ZnTPP) – Critical benchmark molecules in photochemistry and photosynthesis. J. Photochem. Photobiol. C. Photochem. Rev..

[54] Joung JF, Han M, Jeong M, Park S (2020). Experimental database of optical properties of organic compounds. Sci. Data.

[55] Wilkinson MD (2016). Comment: the FAIR guiding principles for scientific data management and stewardship. Sci. Data.

[56] Raccuglia P (2016). Machine-learning-assisted materials discovery using failed experiments. Nature.

[57] Burger B (2020). A mobile robotic chemist. Nature.

[58] MacLeod, B. P. et al. Self-driving laboratory for accelerated discovery of thin-film materials. *Sci. Adv*. **6**, eaaz8867 (2020). 10.1126/sciadv.aaz8867 PMC7220369 32426501

[59] Langner, S. et al. Beyond ternary OPV: high-throughput experimentation and self-driving laboratories optimize multicomponent systems, *Adv. Mater*. **32**, e1907801 (2020). 10.1002/adma.201907801 32049386

[60] Häse F, Roch LM, Kreisbeck C, Aspuru-Guzik A (2018). Phoenics: a Bayesian optimizer for chemistry. ACS Cent. Sci..

[61] Snoek J, Rippel O, Adams RP (2015). Scalable Bayesian optimization using deep neural networks. Proc. PMLR.

[62] Shahriari B, Swersky K, Wang Z, Adams RP, de Freitas N (2016). Taking the human out of the loop: a review of Bayesian optimization. Proc. IEEE.

[63] Wang Z, Zoghi M, Hutter F, Matheson D, de Freitas N (2012). Bayesian optimization in high dimensions via random embeddings. Proc. Twenty-Third Int. Jt. Conf. Artif. Intell..

[64] Krause A, Golovin D (2011). Submodular function maximization. Tractability.

[65] Nakamura T (2022). Selecting molecules with diverse structures and properties by maximizing submodular functions of descriptors learned with graph neural networks. Sci. Rep..

[66] Barelier S (2014). Increasing chemical space coverage by combining empirical and computational fragment screens. ACS Chem. Biol..

[67] Bamborough P, Drewry D, Harper G, Smith GK, Schneider K (2008). Assessment of chemical coverage of kinome space and its implications for kinase drug discovery. J. Med. Chem..

[68] Breiman L (2001). Random forests. Mach. Learn..

[69] Nantasenamat C, Isarankura-Na-Ayudhya C, Naenna T, Prachayasittikul V (2009). A practical overview of quantitative structure-activity relationship. EXCLI J..

[70] Seifrid M (2022). Routescore: punching the ticket to more efficient materials development. ACS Cent. Sci..

[71] Urbina F (2021). UV-adVISor: attention-based recurrent neural networks to predict UV–Vis spectra. Anal. Chem..

[72] Rasmussen, C. E., Williams, C. K. I., Processes, G., *Gaussian Processes for Machine Learning*, M. I. T. Press (2006).

[73] On C, Tanyi EK, Harrison E, Noginov MA (2017). Effect of molecular concentration on spectroscopic properties of poly (methyl methacrylate) thin films doped with rhodamine 6G dye. Opt. Mater. Express.

[74] Choi M-S (2008). One-dimensional porphyrin H-Aggregates induced by solvent polarity. Tetrahedron Lett..

[75] Svetnik V (2003). Random forest: a classification and regression tool for compound classification and QSAR modeling. J. Chem. Inf. Comput. Sci..

[76] Pedregosa F (2011). Scikit-learn: machine learning in python. J. Mach. Learn. Res..

[77] Mauri A, Consonni V, Pavan M, Todeschini R (2006). DRAGON software: an easy approach to molecular descriptor calculations. Match.

[78] Kursa MB, Rudnicki WR (2010). Feature selection with the Boruta package. J. Stat. Softw..

[79] Frisch, M. J. et al. *Gaussian16 Revision C.01*.

