# Supplementary material for: A semi-automated material exploration scheme to predict the solubilities of tetraphenylporphyrin derivatives
Source: Commun Chem. 2022 Nov 22;5:158. doi: 10.1038/s42004-022-00770-9 (PMC9814751; doi:10.1038/s42004-022-00770-9)
Supplement: Supplementary file 1 — Supplementary Information [file 42004_2022_770_MOESM1_ESM.docx]

**Supplementary Information**

**A semi-automated material exploration scheme to predict the solubilities of tetraphenylporphyrin derivatives**

Raku Shirasawa^1*^, Ichiro Takemura^2^, Shinnosuke Hattori^1^, and Yuuya Nagata^3*^

1. Advanced Research Laboratory, R&D Center, Sony Group Corporation, Atsugi Tec. 4-14-1 Asahi-cho, Atsugi-shi, Kanagawa, 243-0014, Japan
2. Tokyo Laboratory 26, R&D Center, Sony Group Corporation, Atsugi Tec. 4-14-1 Asahi-cho, Atsugi-shi, Kanagawa, 243-0014, Japan
3. Institute for Chemical Reaction Design and Discovery, Hokkaido University, Kita 21 Nishi 10, Kita-ku, Sapporo, Hokkaido, 001-0021, Japan

E-mail for Correspondence

Raku Shirasawa; [Raku.Shirasawa@sony.com](mailto:Raku.Shirasawa@sony.com) and Yuuya Nagata; [nagata@icredd.hokudai.ac.jp](mailto:nagata@icredd.hokudai.ac.jp)

**Table of contents**

[**1. Substitution target molecules** S2](#_Toc117587520)

[**2. Mapping molecules over principal components** S3](#_Toc117587521)

[**3. Spectrum analysis** S6](#_Toc117587522)

[**4. Time-dependent density functional theory (TDDFT) calculation** S7](#_Toc117587523)

[**5. Solubility predictions** S8](#_Toc117587524)

[**6. Threshold used in SFMMOL** S18](#_Toc117587525)

[**7. Comparison of algorithms for prediction performances of calculated properties** S19](#_Toc117587526)

[**8. Supplementary Method: Preparation of the top-ranked TPP derivatives 11–15** S27](#_Toc117587527)

# **1. Substitution target molecules**

Supplementary Fig. 1 depicts the substitution target porphyrins from ACCESSIBLE to generate GENERATED.

**Supplementary Fig. 1.** Substitution target molecules from ACCESSIBLE

# **2. Mapping molecules over principal components**

Supplementary Fig. 2 depicts molecular groups (PubChem TPP-all, GENERATED, and ACCESSIBLE) over principal components for all molecules with TPP in PubChem.


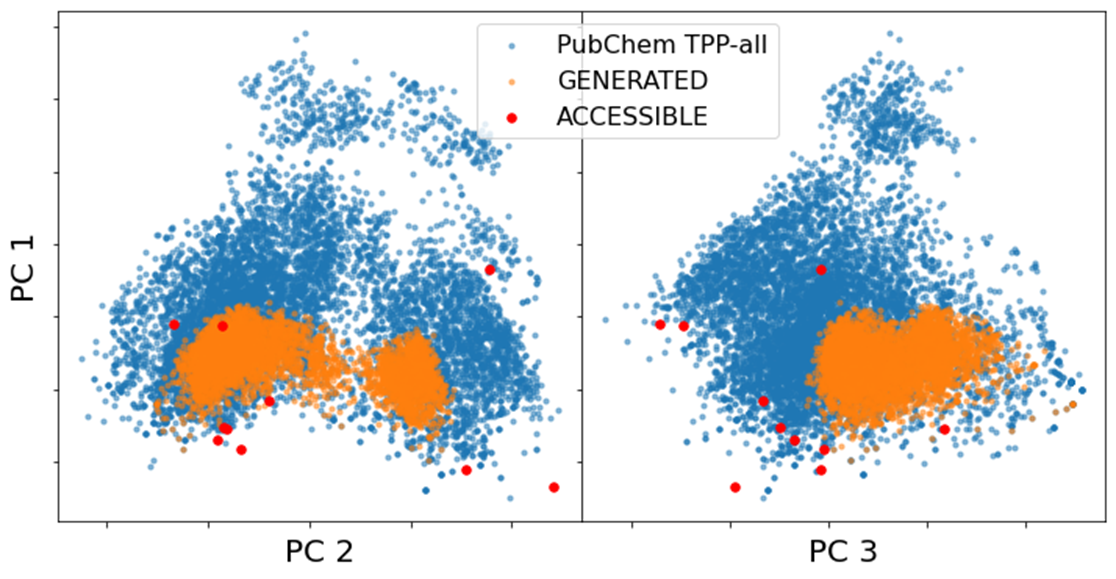


Supplementary Fig. 2. Mapping molecular groups over principal components for all molecules with TPP in PubChem.

Supplementary Fig. 3 depicts molecular groups (PubChem TPP-para, GENERATED, and ACCESSIBLE). TPP-para includes any TPP derivative except for molecules with any substitutions to pyrrole, or any ortho- or meta-substitutions to phenol in the TPP backbone.


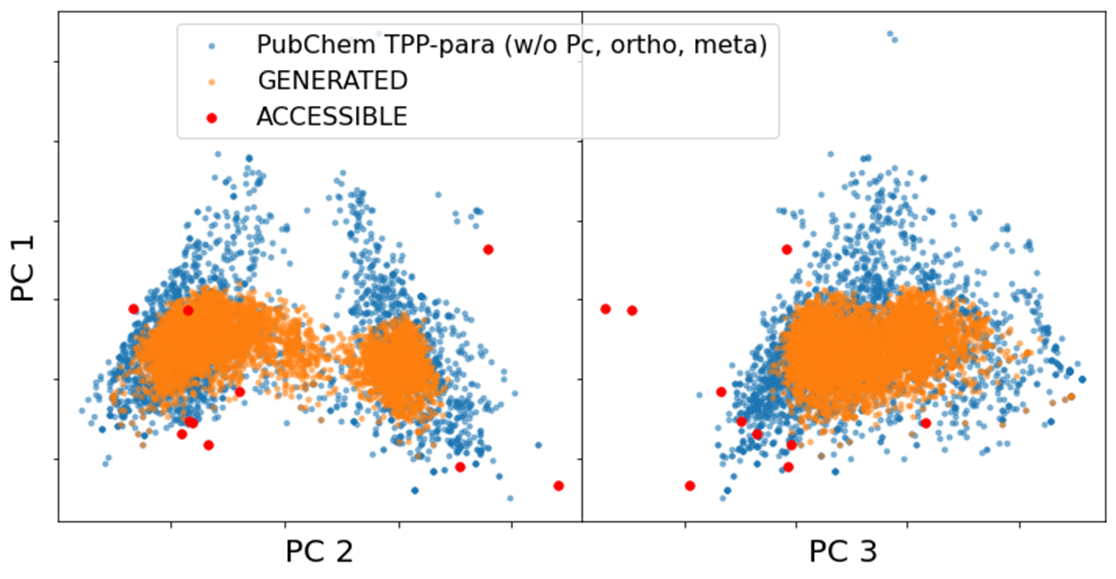


Supplementary Fig. 3. Mapping molecular groups over principal components for all molecules with TPP in PubChem. PubChem TPP-para (w/o Pc, ortho, meta) includes any TPP derivative except for molecules with any substitution to pyrrole or any ortho- or meta-substitution to phenol.

Supplementary Fig. 4 depicts the cumulative contribution rate for principal components (1-10) for all molecules with TPP in PubChem.


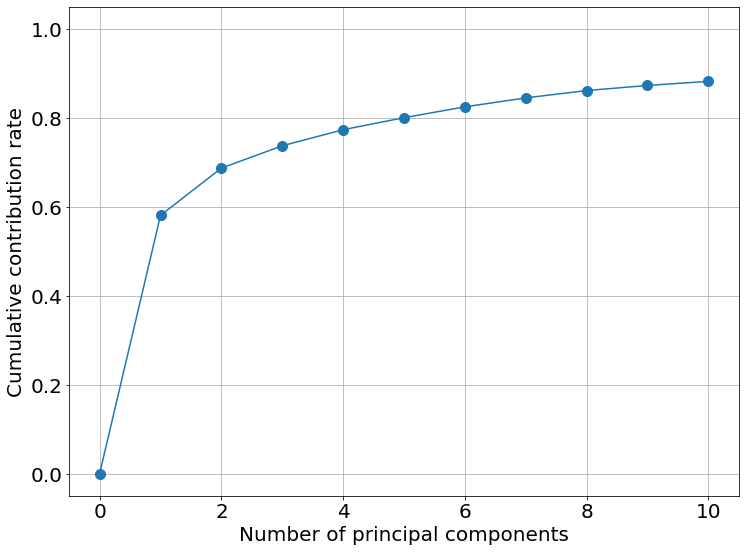


Supplementary Fig. 4. Cumulative contribution rate of principal components for all molecules with TPP in PubChem.

Supplementary Fig. 5 depicts sampled PubChem’s TPP molecules which are not covered by GENERATED. Each molecule in the upper side of the mapping has a large substitution (Molecular Weight (MW) > 200) to a phenyl group, whereas that in the lower side has small substitutions directly to phenyl groups. Larger substituents (MW > 200) or additional reactions, e.g., Suzuki coupling will cover those molecules.


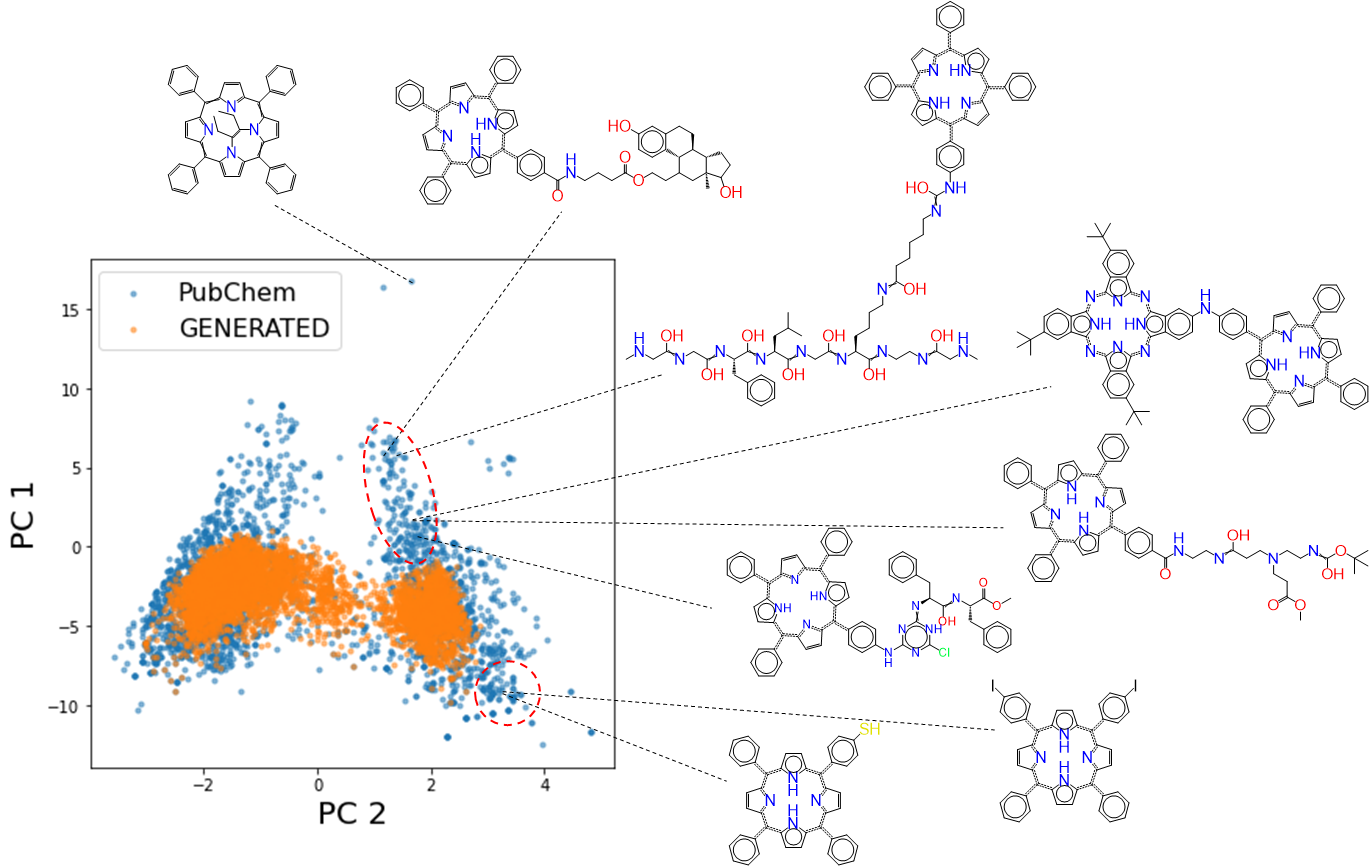


Supplementary Fig. 5. PubChem’s TPP molecules which are not covered by GENERATED are sampled. Each molecule in the upper side of the mapping has a large substitution (Molecular Weight (MW) > 200) to a phenyl group, whereas that in the lower side has small substitutions directly to phenyl groups. Larger substituents (MW > 200) or additional reactions, e.g., Suzuki coupling will cover those molecules.

# **3. Spectrum analysis**

Supplementary Fig. 6a displays the measured spectra for **7** in three solvents: **S4**, **S5**, and **S8**. Supplementary Fig. 6b illustrates schematics to select four indicators for major peaks: λ_max_, Intensity, FWHM, and Area. The first three indicators are calculated from a single-Gaussian fitting, whereas the remaining ones are calculated from a two-Gaussian fitting.


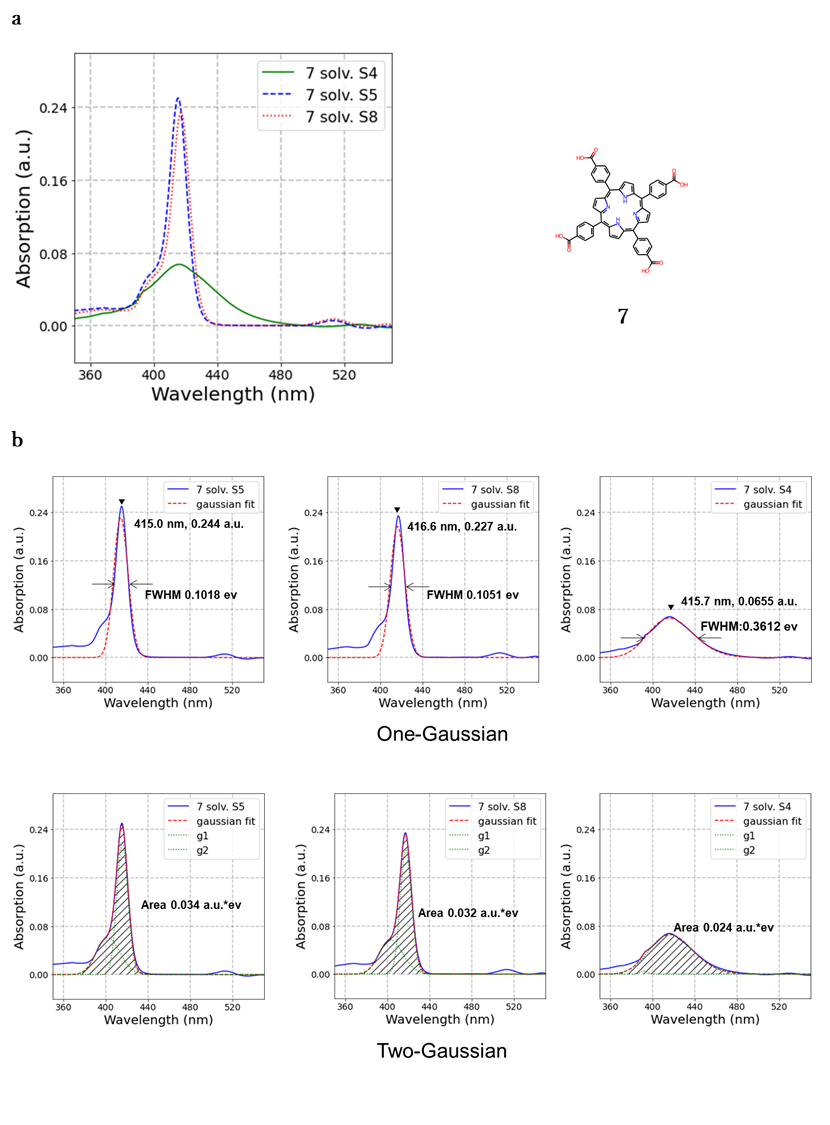


Supplementary Fig. 6. a Measured spectra for 7 in three solvents: S4, S5, and S8. b Schematics to select four indicators for major peaks: λ_max_, Intensity, FWHM, and Area. The first three indicators are calculated from single-Gaussian fitting, whereas the remaining ones are calculated from two-Gaussian fitting.

# **4. Time-dependent density functional theory (TDDFT) calculation**

Supplementary Fig. 7 displays the spectra and their indicators calculated from TDDFT using B3LYP functional with Grimme’s D3BJ dispersion correction and SMD solvent model on Gaussian 16 [1]. Each spectrum represents a convolution of the calculated spectrum with a Gaussian function (σ = 0.45 eV). The top-left chart represents spectra for several molecules with **S5** (methanol), whereas the top-right chart represents spectra for **7** with several solvents. The bottom charts indicate four indicators for the calculated spectra.

**Supplementary Fig. 7.** Spectra and their indicators from TDDFT calculation using B3LYP functional with Grimme’s D3BJ dispersion correction and SMD solvent model on Gaussian 16 [1]. Each spectrum represents a convolution of the calculated spectrum with a Gaussian function (σ = 0.45 eV). The top-left chart represents spectra for several molecules with S5 (methanol), whereas the top-right chart represents spectra for 7 with several solvents. The bottom charts represent four indicators for the calculated spectra.

# **5. Solubility predictions**

Supplementary Table 1 displays categories for each evaluation determined based on FWHM_r_ and Area_r_ calculated in this study; the deep- to light-blue coloured cells denote various categories (0–4).

**Supplementary Table 1.** Categories for each evaluation determined based on FWHM_r_ and Area_r_; deep- to light-blue coloured cells denote various categories (0–4).


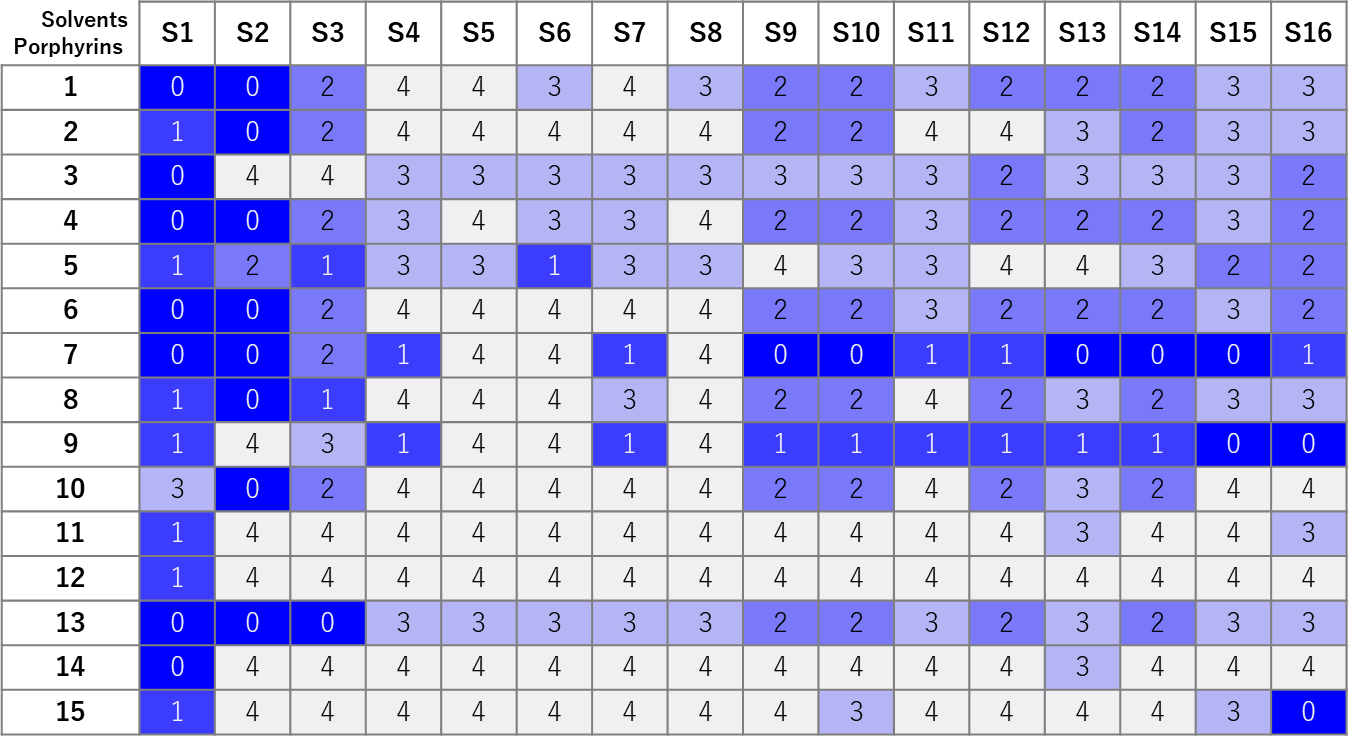


Supplementary Fig. 8 depicts important ECFP bits for a two-class classification developed in this study. **A1** to **A6** belong to porphyrins, whereas **B1** and **B2** pertain to solvents. Blue-hatched atoms represent the centre of each substructure for ECFP calculation. An asterisk (*) represents a nonhydrogen atom. ‘b’ and ‘f’ denote bit number and the frequency of selected important bits, respectively. {**A6**, **A6’**} and {**B1**, **B1’**} represent bit-collision structures.

Supplementary Fig. 8. Important ECFP bits for two-class classification. A1 to A6 pertain to porphyrins, whereas B1 and B2 pertain to solvents. Blue-hatched atoms represent the centre of each substructure for ECFP calculation. Asterisk (*) represents a nonhydrogen atom. ‘b’ and ‘f’ denote bit number and the frequency of selected important bits, respectively. {A6, A6’} and {B1, B1’} represent bit-collision structures.

Supplementary Table 2 depicts the values of three important ECFP bits (**B1**, **B1’** and **B2**) for sixteen solvents. The solvents are divided into five groups (#1–5).

Supplementary Table 2. Values of three important ECFP bits (B1, B1’ and B2) for sixteen solvents. The solvents are divided into five groups (#1–5)

|  | S1 | S2 | S3 | S4 | S5 | S6 | S7 | S8 |
| --- | --- | --- | --- | --- | --- | --- | --- | --- |
| B1 | 1 | 0 | 0 | 0 | 1 | 1 | 0 | 1 |
| B1’ | 0 | 0 | 1 | 0 | 0 | 0 | 1 | 0 |
| B2 | 0 | 1 | 1 | 1 | 1 | 1 | 1 | 1 |
| Group | #1 | #2 | #2 | #2 | #4 | #4 | #3 | #4 |
|  |  |  |  |  |  |  |  |  |
|  | **S9** | **S10** | **S11** | **S12** | **S13** | **S14** | **S15** | **S16** |
| B1 | 0 | 0 | 0 | 0 | 0 | 0 | 0 | 0 |
| B1’ | 0 | 0 | 0 | 0 | 0 | 0 | 0 | 0 |
| B2 | 0 | 0 | 0 | 0 | 1 | 0 | 0 | 1 |
| Group | #5 | #5 | #5 | #5 | #2 | #5 | #5 | #2 |

Supplementary Table 3 and 4 depict accuracies of the classification models with ECFP for training and test data, respectively.

Supplementary Table 3. Accuracies of the classification models with ECFP for training data.


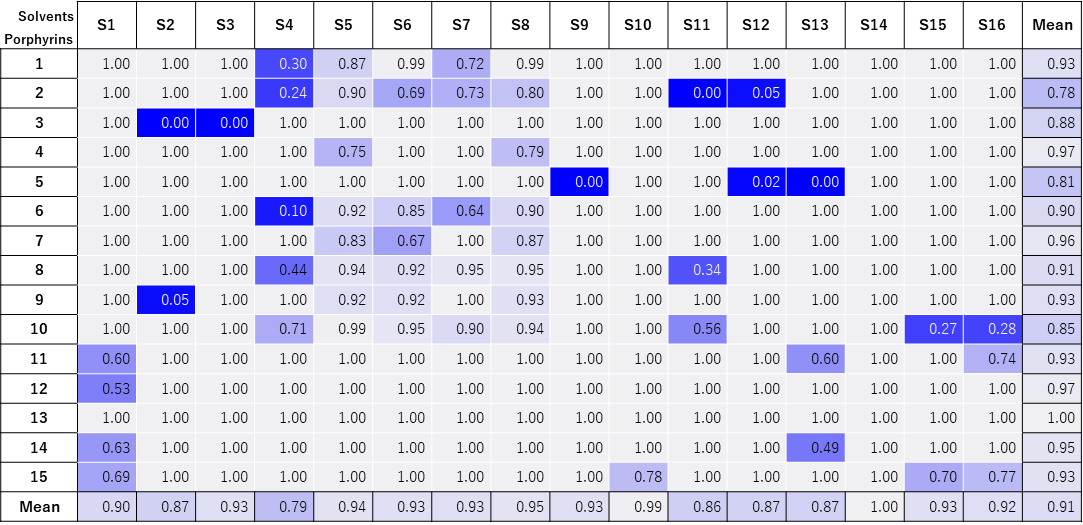


Supplementary Table 4. Accuracies of the classification models with ECFP for test data.


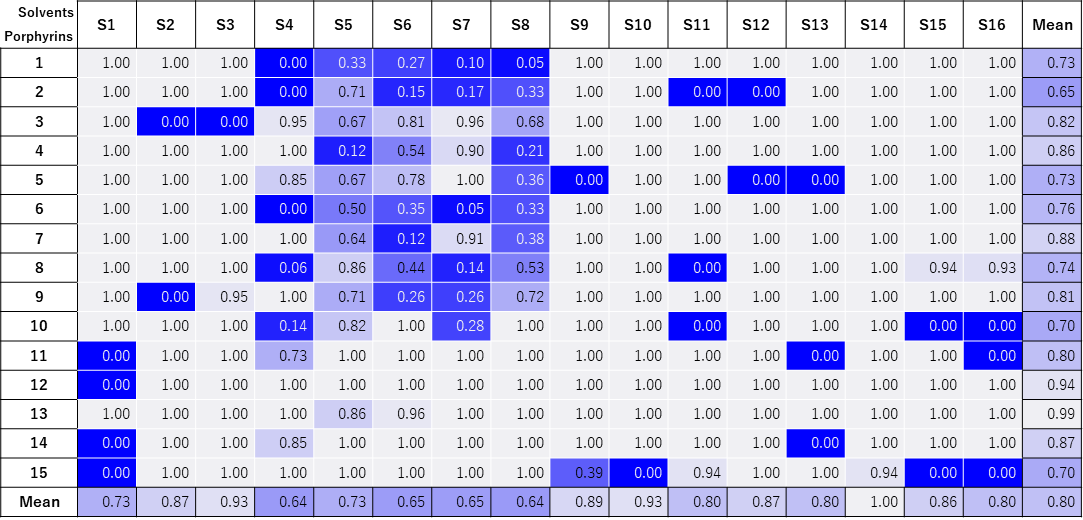


Supplementary Table 5 presents important DRAGON descriptors for the two-class classification developed in this study. The prefix ‘SLV’ represents the descriptor pertaining to solvents; ‘Freq.’ represents the frequency of ‘important’ selections. Descriptions are quoted from the Dragon7 manual.

**Supplementary Table 5.** Important DRAGON descriptors for two-class classification. The prefix ‘SLV’ represents the descriptor pertaining to solvents; ‘Freq.’ represents the frequency of ‘important’ selections. Descriptions are quoted from the Dragon7 manual.

| **#** | **Descriptor name** | **Freq.** | **Description** |
| --- | --- | --- | --- |
| **1** | AMW | 1.0 | average molecular weight |
|  | H% | 1.0 | percentage of H atoms |
|  | P_VSA_LogP_1 | 1.0 | P_VSA-like on LogP, bin 1 |
|  | P_VSA_LogP_7 | 1.0 | P_VSA-like on LogP, bin 7 |
|  | SLV_MW | 1.0 | (solv.) molecular weight |
| **6** | Mv | 0.98 | mean atomic van der Waals volume |
|  | SLV_GD | 0.98 | (solv.) graph density |
| **8** | SLV_MLOGP2 | 0.97 | (solv.) squared Moriguchi octanol-water partition coef. |
|  | SLV_PDI | 0.97 | (solv.) packing density index |
| **10** | SLV_ALOGP2 | 0.90 | (solv.) squared Ghose-Crippen octanol-water |
| **11** | nH | 0.89 | number of Hydrogen atoms |
| **12** | MW | 0.87 | molecular weight |
| **13** | Hy | 0.86 | hydrophilic factor |
| **14** | SLV_Vx | 0.83 | (solv.) McGowan volume |
| **15** | P_VSA_s_3 | 0.76 | P_VSA-like on I-state, bin 3 |
|  | SLV_TPSA(NO) | 0.76 | (solv.) topological polar surface area using N,O |
| **17** | N% | 0.67 | percentage of N atoms |
| **18** | SLV_AMR | 0.63 | (solv.) Ghose-Crippen molar refractivity |
| **19** | SLV_P_VSA_ppp_D | 0.61 | (solv.) P_VSA-like on potential pharmacophore |
| **20** | SLV_P_VSA_s_6 | 0.53 | (solv.) P_VSA-like on I-state, bin 6 |
| **21** | SLV_P_VSA_v_2 | 0.52 | (solv.) P_VSA-like on van der Waals volume, bin 2 |

Supplementary Table 6 and 7 depict accuracies of the classification models with DRAGON for training and test data, respectively.

Supplementary Table 6. Accuracies of the classification models with DRAGON for training data.


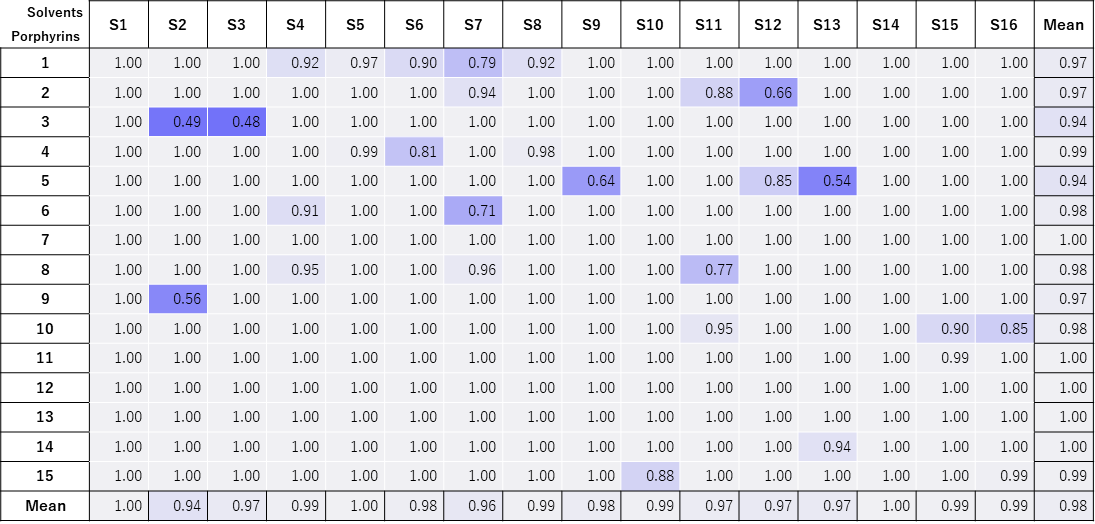


Supplementary Table 7. Accuracies of the classification models with DRAGON for test data.


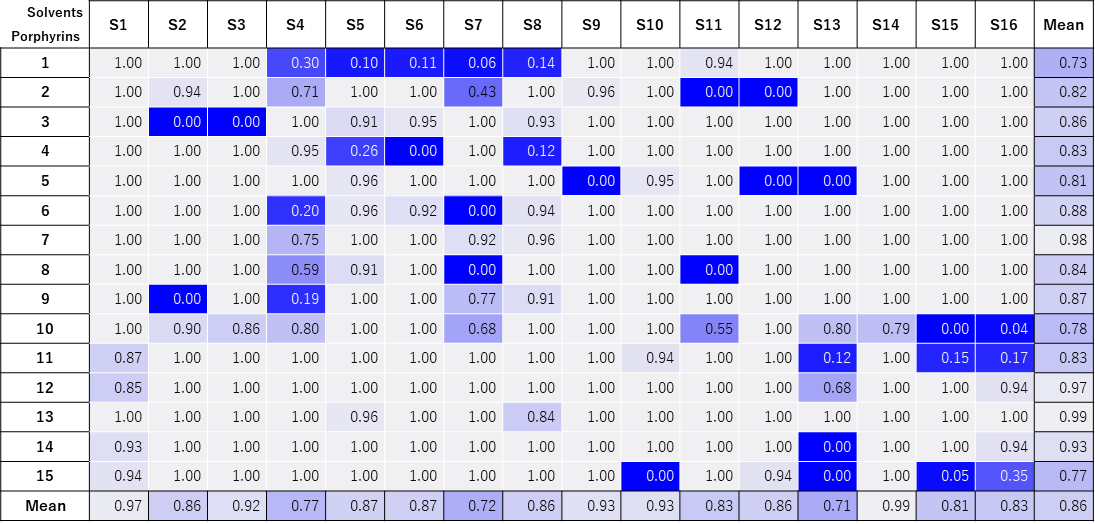


We used Scikit-learn [2] to develop prediction models and determine their performance (accuracy, balanced accuracies, R^2^, and MAE) except for the Hansen solubility parameters (HSP). Supplementary Fig. 9 displays schematics of the construction of ML models. For both inputs (ECFP and DRAGON), each input vector is a concatenation of two vectors: a vector for porphyrin and a vector for the solvent.


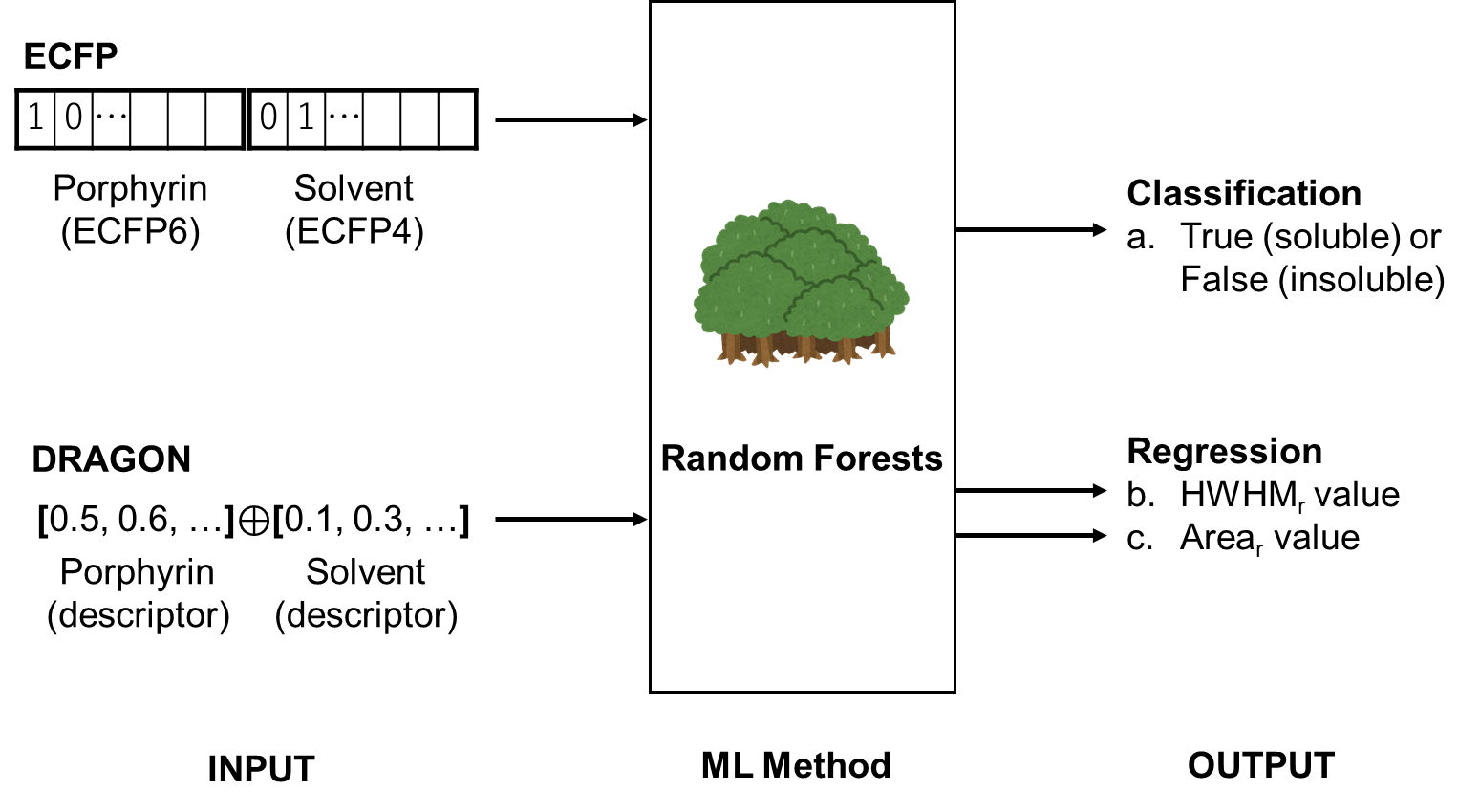


Supplementary Fig. 9. Schematics of construction of ML models. For both inputs (ECFP and DRAGON), each input vector is a concatenation of two vectors: a vector for porphyrin and solvent, each.

Supplementary Table 8 depicts solubility related to the relative energy difference (RED) of estimated HSP. Supplementary Table 9 depicts the HSP solubility in binary data settings with a RED threshold (=0.741) having an experimental ratio of good solvents (0) : poor solvents (1) = 96 : 144. Supplementary Table 10 depicts a comparison between the estimated binary HSP solubility and the experiment. The accuracy is 0.45, which is lower than that of our classifier (0.80 or 0.87).

**Supplementary Table 8.** Relative energy difference (RED) of estimated Hansen solubility parameters (HSPs) for each pair of molecule and solvent. RED>1 indicates that the pair is insoluble, RED~1 indicates that the pair is partially soluble, and RED<1 indicates that the pair is soluble, respectively. Cells are coloured by RED values.

**Supplementary Table 9.** HSP solubility in binary data setting under a RED threshold (=0.741) having the experimental ratio of good solvents (0) : poor solvents (1) as 96 : 144.

**Supplementary Table 10.** Comparison between the estimated binary HSP solubility and the experiment. The accuracy is 0.45, which is lower than that of our classifier (0.80 or 0.87).

Supplementary Table 11 depicts five-class classification (0-4) based on ECFP or DRAGON by AutoML (auto-sklearn).

**Supplementary Table 11**. Five-class classification based on ECFP or DRAGON by AutoML (auto-sklearn). We use Scikit-learn [2] to estimate the models and calculate the balanced accuracies.

| Variable set | Balanced Accuracy | |
| --- | --- | --- |
|  | Train | Test |
| ECFP | 0.77 | 0.69 |
| DRAGON | 0.82 | 0.72 |

Supplementary Fig. 10 depicts the predictions and observations of FWHM_r_ and Area_r_ from ECFP or DRAGON; horizontal and vertical axes represent the observed and predicted values, respectively. Supplementary Table 12 shows the Regression of FWHM_r_ and Area_r_ without solvent **S1** (Water) by AutoML (auto-sklearn).

Supplementary Fig. 10. Predictions of FWHM_r_ and AREA_r_ from ECFP or DRAGON; horizontal and vertical axes represent the observed and predicted values, respectively.

**Supplementary Table 12.** Regression of FWHM_r_ and Area_r_ without solvent **S1** (Water) by AutoML (auto-sklearn). R^2^ and MAE are the coefficient of determination (implemented in Scikit-learn [2]) and mean absolute error, respectively.

| Variable set | FWHM_r_ R^2^ | | FWHM_r_ MAE | | Area_r_ R^2^ | | Area_r_ MAE | |
| --- | --- | --- | --- | --- | --- | --- | --- | --- |
|  | Train | Test | Train | Test | Train | Test | Train | Test |
| ECFP | 0.70 | 0.56 | 0.093 | 0.12 | 0.88 | 0.67 | 0.067 | 0.14 |
| DRAGON | 0.88 | 0.020 | 0.052 | 0.19 | 0.86 | 0.55 | 0.080 | 0.15 |

# **6. Threshold used in SFMMOL**

**Supplementary Note 1.** The threshold ($D_{\mathrm{TH}}$) can be set from 0 to 1.0, as the Tanimoto distance is defined as same value range. Using the threshold, MOLSPACE can be described as network (graph), wherein a node represents a molecule in MOLSPACE and an edge between two nodes represents their distance < $D_{\mathrm{TH}}$. Supplementary Fig. 11 depicts four network characteristics–number of total edges, clustering coefficient, number of connected components, and mean number of edges for each node–for $D_{\mathrm{TH}}$ values varying from 0.1 to 0.9. For $D_{\mathrm{TH}} \geq0.5$, most nodes connect with each other, making dense graphs (number of connected components $\leq$ 4, mean number of edges for each node > 600, and clustering coefficient (indicator for denseness) > 0.69). Moreover, handling a large number of total edges (> 1.7 Mil.) is very demanding in terms of both computation and memory footprint; thus, those large values are not suitable for $D_{\mathrm{TH}}$. By contrast, for $D_{\mathrm{TH}} \leq0.2,$ most nodes are too isolated to be covered by other nodes in $\mathrm{MOLSPACE}$ (number of total edges < $\mathrm{Count}\left( \mathrm{MOLSPACE} \right)=5910$). Therefore, the threshold should be set in the range of $0.2< D_{\mathrm{TH}}<0.5$. As stated in the main text, we selected $D_{\mathrm{TH}}=0.3$ to calculate coverage, which is a sparsely connected case (mean number of edges for each node = 9.4). There is a more densely connected option $D_{\mathrm{TH}}=0.4$ (mean number of edges for each node = 78.5) that produces larger values for the coverage calculation.

Supplementary Fig. 11. Four characteristics (top-left: number of total edges, top-right: clustering coefficient, bottom-left: number of connected components, and bottom-right: mean number of edges for each node) of the MOLSPACE network for threshold ($\boldsymbol{D}_{\mathbf{TH}}$) values varying from 0.1 to 0.9.

# **7. Comparison of algorithms for prediction performances of calculated properties**

**Supplementary Note 2.** The searching algorithms (SFMMOL), random sampling (RANDOM), and BO-like uncertainty sampling (UNC) were compared in terms of their performance with respect to prediction models obtained from a selected molecular set. To estimate and evaluate the ML models, we utilised random forest regression (RFR) as the ML method, ECFP6 as explanatory variables (INPUT), and three calculated descriptors of molecular properties implemented in RDKit (https://www.rdkit.org) as objective variables (OUTPUT) : VSA_EState1, SlogP_VSA1, and MolMR. Supplementary Figs. 12–14 depict the comparisons of the selection methods regarding coverage (a) and ML-model performances (R2 (b), MAE (c), and relative MAE (d)) for the prediction of calculated properties (VSA_EState1, SlogP_VSA1, and MolMR) implemented on RDKit (https://www.rdkit.org). The coverages of SFMMOLs rapidly increased in the early stage (NoE < 500) and reached to 80 %; they were 10–20 % (20–30 %) larger than that of RANDOM (UNC). Ignoring the low performance regions (R^2^ < 0), SFMMOLs with large λ (= 1 or 0.01) exhibited the best performances in terms of MAE for the prediction of VSA_EStage1 and SlogP_VSA1, whereas RANDOM presented the best MAE for the prediction of MolMR. SFMMOLs with large λ (= 1 or 0.01) mostly produced a better MAE than SFMMOLs with small λ (= 0, 0.001, or 0.0002). Overall, SFMMOLs presented the best performance in terms of coverage, whereas SFMMOLs with large λ or RANDOM exhibited the best performance in terms of MAE. As stated in the main text, we used SFMMOL (λ = 1) to select the molecular order of evaluation.


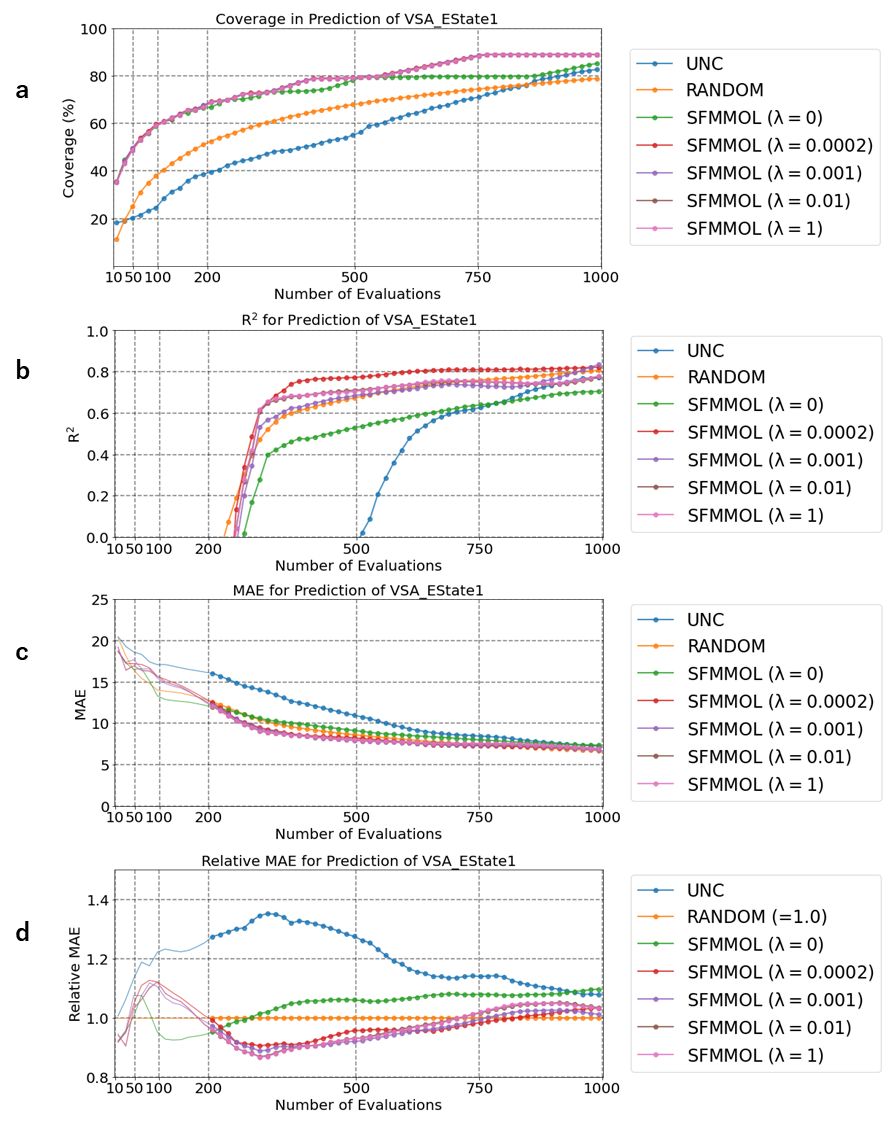


Supplementary Fig. 12. Comparison of selection methods in terms of coverage (a) and ML-model performance (R2 (b), MAE (c), and relative MAE (d)) for the prediction of calculated property VSA_EState1 implemented on RDKit (https://www.rdkit.org). The ML models were estimated for each evaluation using the training datasets selected by each method. The coverages of SFMMOLs rapidly increased in the early stage (number of evaluations (NoE) < 500) and reached 80 %; they were 10–20 % (20–30 %) larger than that of RANDOM (UNC). Ignoring the low performance region (R^2^ < 0 and NoE < 200), SFMMOLs (λ > 0) exhibited better performances in terms of MAE until NoE < 700, wherein the relative MAE is 0.87–1.0. SFMMOL (λ = 0) presented a larger MAE than RANDOM in NoE > 300. SFMMOLs with large λ (≥ 0.01) produced best MAEs in NoE < 400, whereas SFMMOLs with small λ (≤ 0.001) produced best MAEs in 400 ≤ NoE ≤ 800.


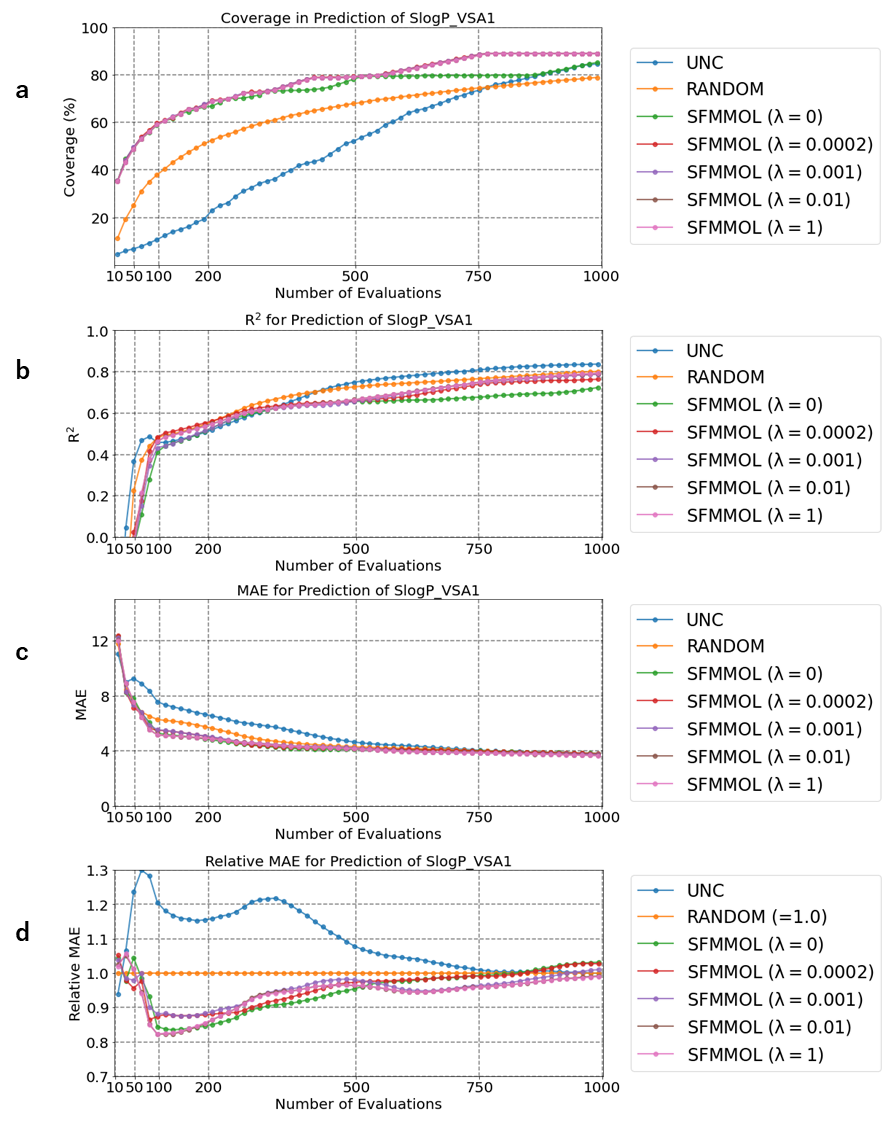


Supplementary Fig. 13. Comparison of selection methods in terms of coverage (a) and ML-model performance (R2 (b), MAE (c), and relative MAE (d)) for the prediction of calculated property SlogP_VSA1 implemented on RDKit (https://www.rdkit.org). The ML models were estimated for each evaluation using the training datasets selected by each method. The coverages of SFMMOLs rapidly increased in the early stage (NoE < 500) and reached 80 %; they were 10–20 % (20–30 %) larger than that of RANDOM (UNC). Ignoring the low performance region (R^2^ < 0 and NoE < 50), SFMMOLs exhibited better performances in terms of MAE until NoE < 800, wherein the relative MAE is in 0.82–1.0. SFMMOLs with small λ (≤ 0.0002) presented a slightly larger MAE than RANDOM in NoE ≥ 800. SFMMOLs (λ = 1 or 0.01) produced the best relative MAE (0.82) at NoE = 96.


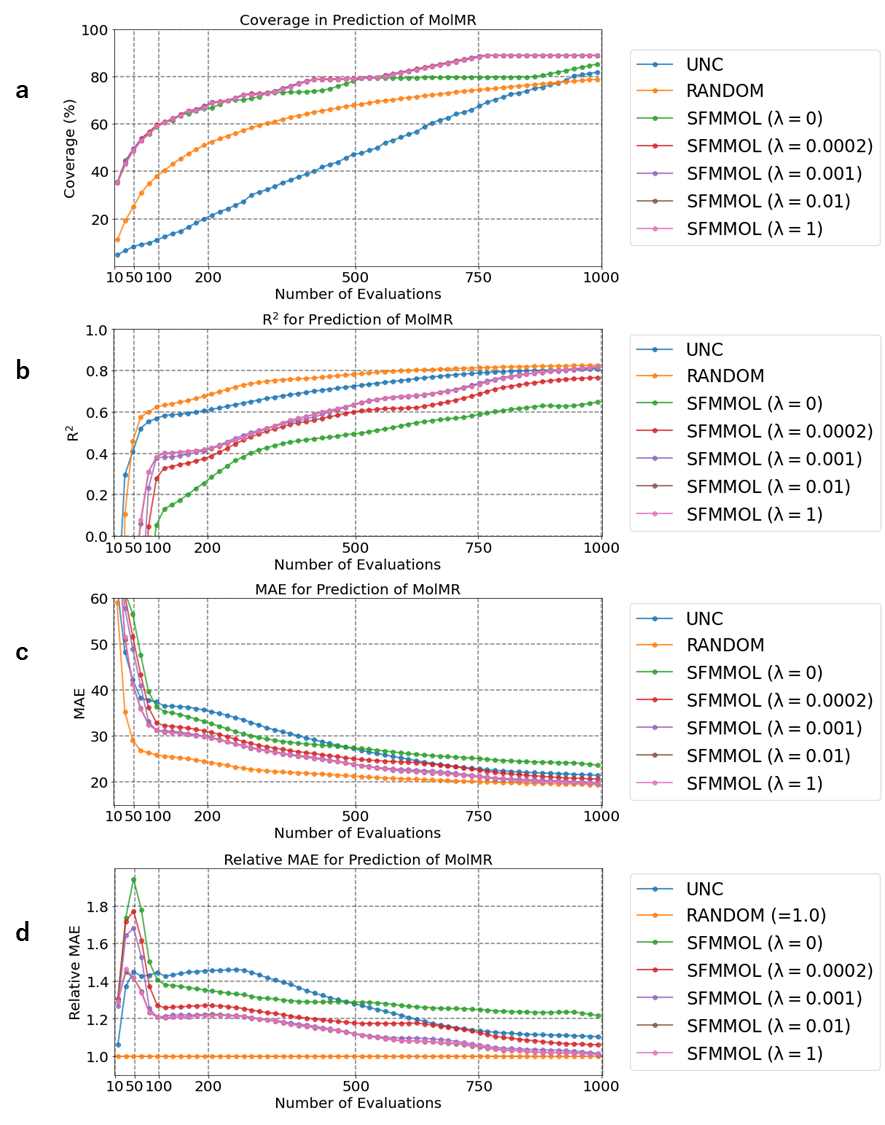


Supplementary Fig. 14. Comparison of selection methods in terms of coverage (a) and ML-model performance (R2 (b), MAE (c), and relative MAE (d)) for the prediction of calculated property MolMR implemented on RDKit (https://www.rdkit.org). The ML models were estimated for each evaluation using the training datasets selected by each method. The coverages of SFMMOLs rapidly increased in the early stage (NoE < 500) and reached 80 %; they were 10–20 % (20–30 %) larger than that of RANDOM (UNC). Ignoring the low performance region (R^2^ < 0 and NoE < 50), RANDOM exhibited the best performance in terms of MAE. In 100 < NoE < 1000, SFMMOLs with large λ (= 1, 0.01, or 0.001) presented the second best MAEs; the relative MAEs were 1.0–1.2. On the other hand, in the same region, SFMMOLs with small λ (= 0.0002 or 0) presented larger MAEs; the relative MAEs were 1.1–1.4.

Supplementary Fig. 15–17 depict the comparisons of simple random sampling (RANDOM), stratified random sampling (stratum is selected by five base structures 4, 5, 6, 7 and 9: SR (5-Bases)), and stratified random sampling (stratum is selected by KNN for ECFP: SR (5-Means)) in terms of coverage (a) and ML-model performance (R2 (b) and MAE (c)) for the prediction of calculated property VSA_Estate1, SlogP_VSA, and MolMR implemented on RDKit (https://www.rdkit.org). Each random sampling method is repeated for ten times; line charts depict the means, and vertical lines depict the standard deviations. Although little difference exists in the early stage, there was no significant difference in the sampling methods.


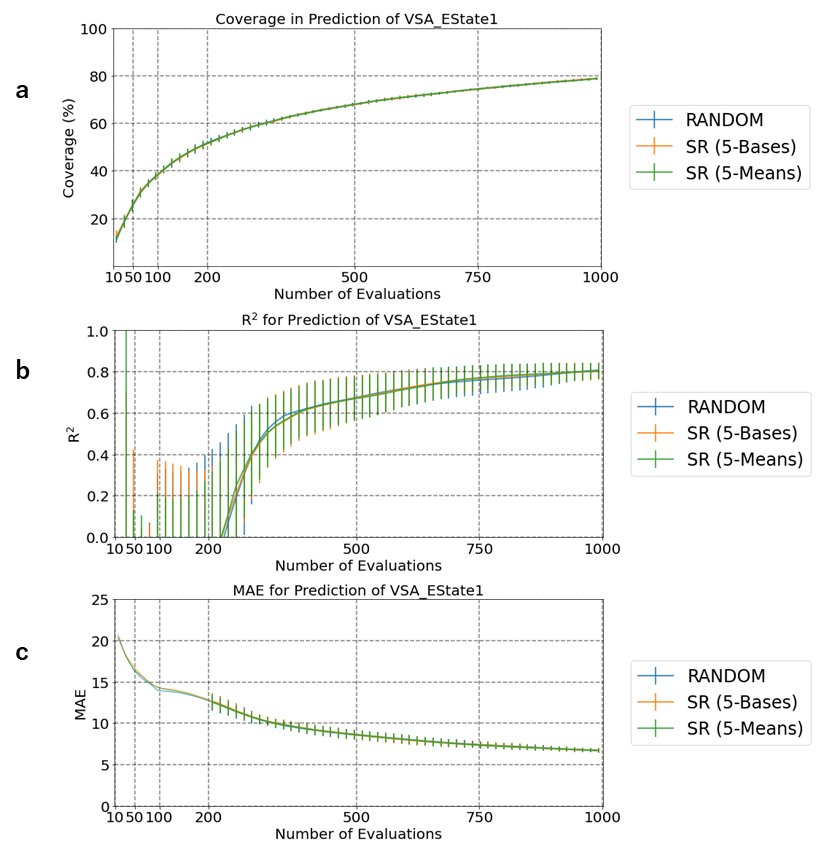


Supplementary Fig. 15. Comparison of simple random sampling (RANDOM), stratified random sampling (stratum is selected by five base structures 4, 5, 6, 7 and 9: SR (5-Bases)), and stratified random sampling (stratum is selected by KNN for ECFP: SR (5-Means)) in terms of coverage (a) and ML-model performance (R2 (b) and MAE (c)) for the prediction of calculated property VSA_Estate1 implemented on RDKit (https://www.rdkit.org). Each random sampling method is repeated for ten times; line charts depict the means, and vertical lines depict the standard deviations. Although little difference exists in early stage (Number of Evaluations < 200), no significant difference in the sampling methods.


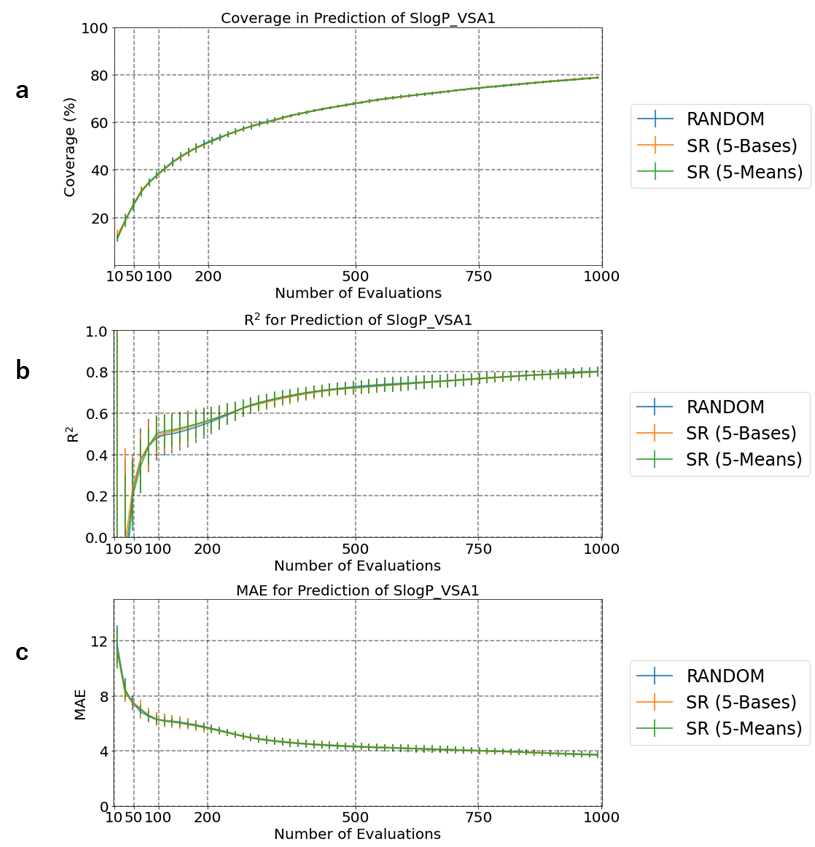


Supplementary Fig. 16. Comparison of simple random sampling (RANDOM), stratified random sampling (stratum is selected by five base structures 4, 5, 6, 7 and 9: SR (5-Bases)), and stratified random sampling (stratum is selected by KNN for ECFP: SR (5-Means)) in terms of coverage (a) and ML-model performance (R2 (b) and MAE (c)) for the prediction of calculated property SlogP_VSA1 implemented on RDKit (https://www.rdkit.org). Each random sampling method is repeated for ten times; line charts depict the means, and vertical lines depict the standard deviations. Although little difference exists in early stage (Number of Evaluations < 100), no significant difference in the sampling methods.


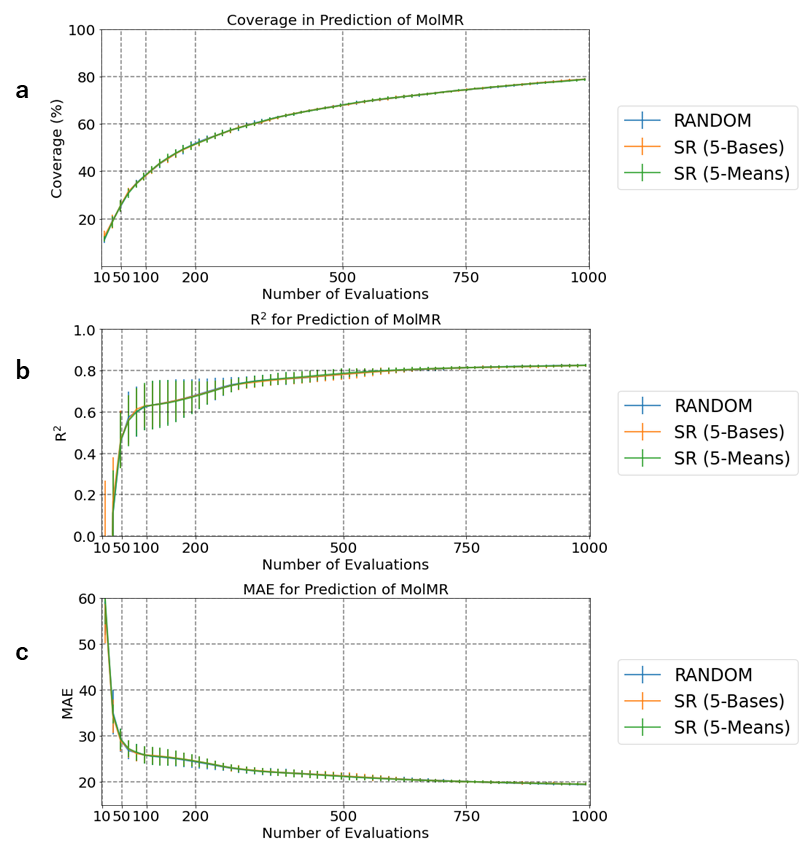


Supplementary Fig. 17. Comparison of simple random sampling (RANDOM), stratified random sampling (stratum is selected by five base structures 4, 5, 6, 7 and 9: SR (5-Bases)), and stratified random sampling (stratum is selected by KNN for ECFP: SR (5-Means)) in terms of coverage (a) and ML-model performance (R2 (b) and MAE (c)) for the prediction of calculated property MolMR implemented on RDKit (https://www.rdkit.org). Each random sampling method is repeated for ten times; line charts depict the means, and vertical lines depict the standard deviations. Although little difference exists in early stage (Number of Evaluations < 50), no significant difference in the sampling methods.

# **8. Supplementary Method: Preparation of the top-ranked TPP derivatives 11–15**

All reactions were carried out under an atmosphere of nitrogen with magnetic stirring. ^1^H (400 MHz) and ^13^C (100 MHz) NMR spectra were recorded on a JEOL ESZ-400S spectrometer at ambient temperature. ^1^H NMR spectra were reported as follows; chemical in ppm downfield from tetramethylsilane (δ scale) relative to CHCl_3_ in CDCl_3_ (7.26 ppm), multiplicity (s = singlet, d = doublet, t = triplet, m = multiplet, and br = broad), coupling constant (Hz), and integration. All ^13^C NMR (100 MHz) spectra were obtained with complete proton decoupling, and the chemical shifts in ppm downfield from tetramethylsilane (δ scale) relative to CHCl_3_ (77 ppm). Fourier transform infrared (FT-IR) spectra were recorded on a JASCO FT/IR-4600ST FT-IR spectrometer equipped with JASCO ATR PRO ONE attenuated total reflection (ATR) attachment with a ZnSe prism. High-resolution mass spectra were obtained with a Thermo Fisher. SCIENTIFIC EXACTIVE spectrometer with electrospray ionization (ESI) mode in Instrumental Analysis Division, Global Facility Center, Creative Research Institution, Hokkaido University. Column chromatography on silica gel was performed on a Biotage Isolera One ISO-1SV Spektra equipped with Biotage Rening Cartridges (10, 30, or 45 g). Tetrahydrofuran (THF) was dried and deoxygenized using an alumina/catalyst column system (Glass Contour Co.). Other chemical reagents were purchased from commercial sources and were used without further purification.

**Synthesis of propyl 4-(10,15,20-triphenylporphyrin-5-yl)benzoate (11)**

5-(4-Carboxyphenyl)-10,15,20-triphenylporphyrin (30.0 mg, 0.046 mmol), 1-(3-dimethylaminopropyl)-3-ethylcarbodiimide hydrochloride (21.8 mg, 0.0218 mmol, 2.5 eq), 4-dimethylaminopyridine (13.9 mg, 0.114 mmol, 2.5 eq), and 1-propanol (17.0 μL, 13.7 mg, 0.228 mmol, 5 eq) were dissolved in tetrahydrofuran (1.8 mL) and stirred for 18 hours at 40 °C. After the reaction, all volatiles were removed in vacuo and the residue was diluted with dichloromethane (4 mL) and washed with water (4 mL). The organic layer was dried with Na_2_SO_4_, and then all volatiles were removed in vacuo. The residue was purified with silica gel flash column chromatography (*n*-hexane/AcOEt = 90/10) to give **11** as a purple solid (19.3 mg, 60%).

^1^H-NMR (CDCl_3_) δ 8.86 (2H, d, *J* = 7.6 Hz), 8.85 (4H, s), 8.80 (2H, d, *J* = 4.8 Hz), 8.45 (2H, d, *J* = 7.6 Hz), 8.31 (2H, d, *J* = 8.0 Hz), 8.23 (3H, d, *J* = 1.2 Hz), 8.21 (3H, d, *J* = 2.0 Hz), 7.81–7.73 (9H, m), 4.48 (2H, t, *J* = 6.6 Hz), 2.00–1.91 (2H, m), 1.17 (3H, t, *J* = 7.4 Hz), –2.79 (2H, s). ^13^C-NMR (CDCl_3_) 166.9, 146.9, 142.0, 134.5, 131.3 (br s, α and β positions of pyrrole, and the *meso* position of the porphyrin ring were overlapped), 129.9, 127.9, 127.8, 126.7, 120.5, 120.3, 118.6, 66.9, 22.3, 10.7. FT-IR (ATR, ZnSe) 3314, 3053, 2958, 2920, 2851, 2706, 2606, 2533, 1944, 1809, 1717, 1605, 1596, 1558, 1490, 1472, 1439, 1399, 1375, 1348, 1308, 1268, 1221, 1175, 1153, 1096, 1071, 1031, 1020, 1000, 980, 964, 938, 904, 876, 865 cm^–1^. HRMS (ESI) m/z calcd for C_48_H_37_N_4_O_2_ (M+H^+^): 701.29165, found: 701.29277.


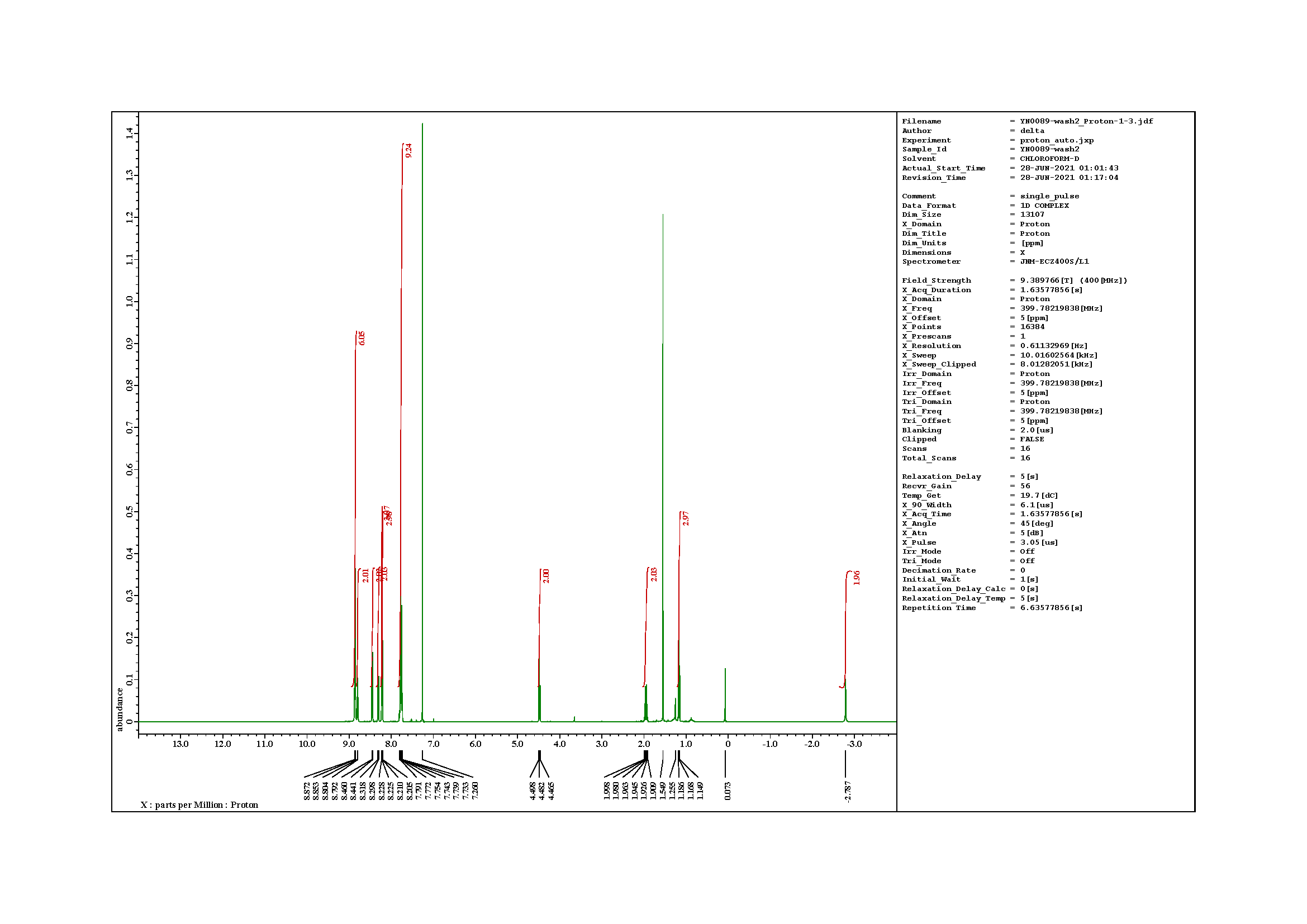


**Supplementary Fig. 18.** ^1^H NMR spectrum of **11** in CDCl_3_ at room temperature.


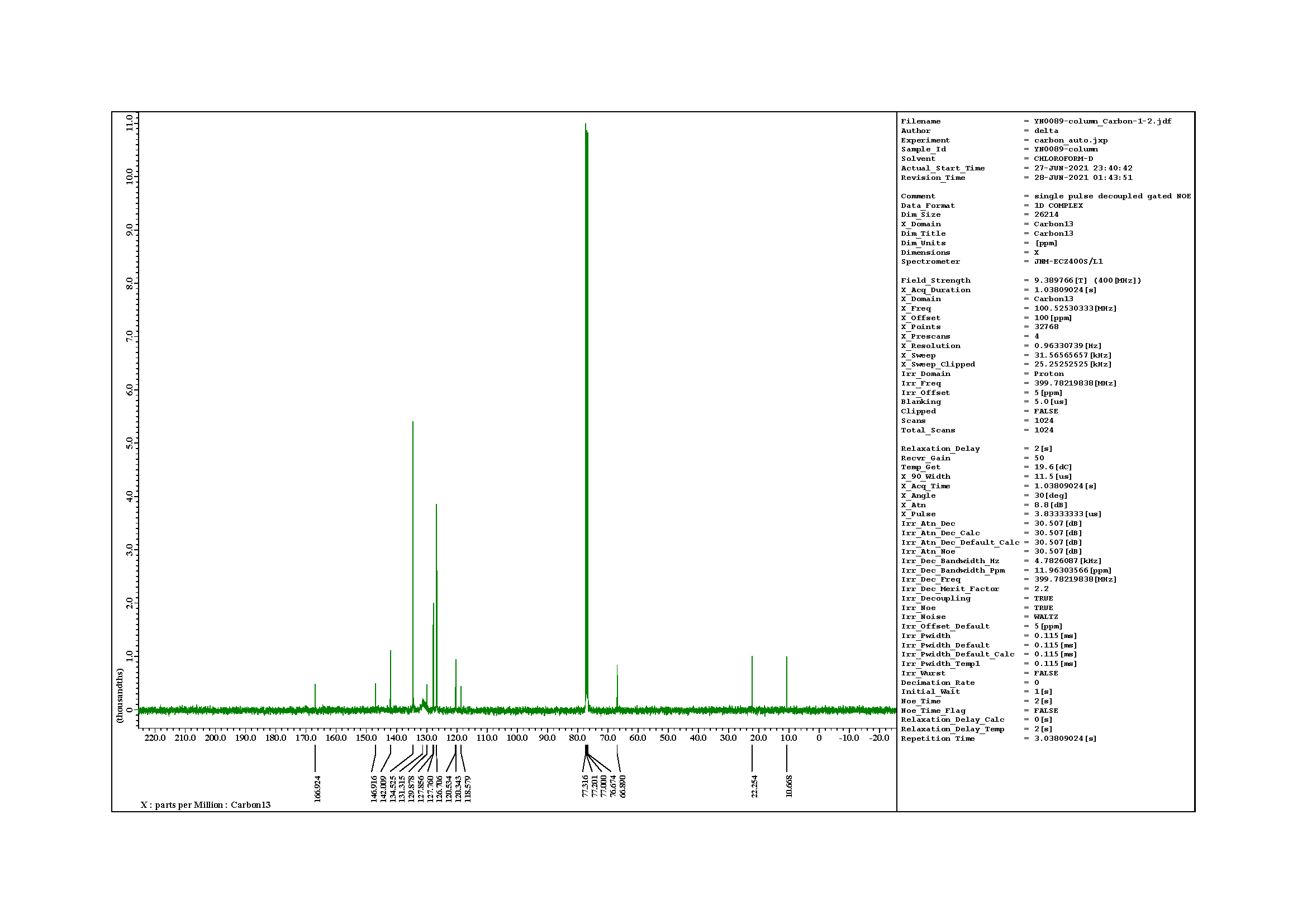
 **Supplementary Fig. 19.** ^13^C NMR spectrum of **11** in CDCl_3_ at room temperature.

**Synthesis of tetrabutyl 2,2',2'',2'''-((porphyrin-5,10,15,20-tetrayltetrakis(benzene-4,1-diyl))
tetrakis(oxy))tetraacetate (12)**

5,10,15,20-Tetrakis(4-carboxymethyloxyphenyl)porphyrin (40.0 mg, 0.061 mmol), 1-(3-dimethylaminopropyl)-3-ethylcarbodiimide hydrochloride (58.2 mg, 0.304 mmol, 5 eq), 4-dimethylaminopyridine (37.1 mg, 0.304 mmol, 5 eq), and 1-butanol (55.6 μL, 45.0 mg, 0.607 mmol, 10 eq) were dissolved in *N*,*N*’-dimethylformamide (1.2 mL) and stirred for 36 hours at room temperature. After the reaction, all volatiles were removed in vacuo. The residue was purified with silica gel flash column chromatography (*n*-hexane/AcOEt = 80/20) to give **12** as a purple solid (19.4 mg, 28%).

^1^H-NMR (CDCl_3_) δ 8.85 (s, 8H), 8.13 (d, *J* = 8.6 Hz, 8H), 7.30 (d, *J* = 9.2 Hz, 8H), 4.93 (s, 8H), 4.36 (d, *J* = 13.4 Hz, 8H), 1.80–1.73 (m, 8H), 1.53–1.43 (m, 8H), 1.01 (t, *J* = 7.3 Hz, 12H), –2.79 (s, 2H). ^13^C-NMR (CDCl_3_) δ 169.2, 157.7, 135.6, 131.0 (br s, α and β positions of pyrrole, and the *meso* position of the porphyrin ring were overlapped), 119.5, 112.9, 65.6, 65.4, 30.6, 19.1, 13.7. FT-IR (ATR, ZnSe) 3313, 3030, 2957, 2930, 2871, 1757, 1730, 1603, 1575, 1557, 1503, 1470, 1428, 1408, 1378, 1349, 1281, 1235, 1200, 1173, 1108, 1082, 1025, 991, 981, 965, 879, 840, 796, 735, 709 cm^–1^. HRMS (ESI) m/z calcd for C_68_H_71_N_4_O_12_ (M+H^+^): 1135.50685, found: 1135.50761.


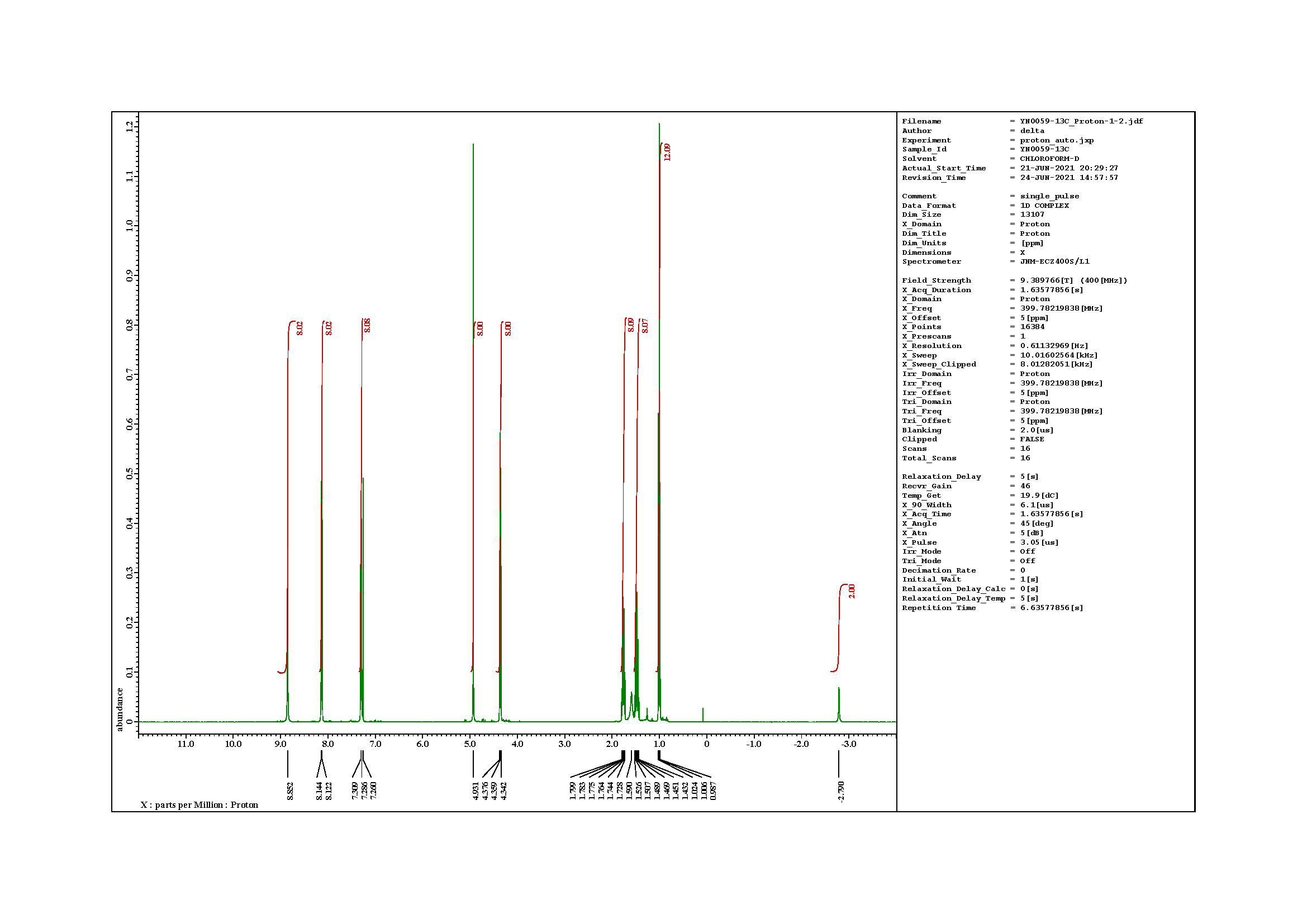
 **Supplementary Fig. 20.** ^1^H NMR spectrum of **12** in CDCl_3_ at room temperature.


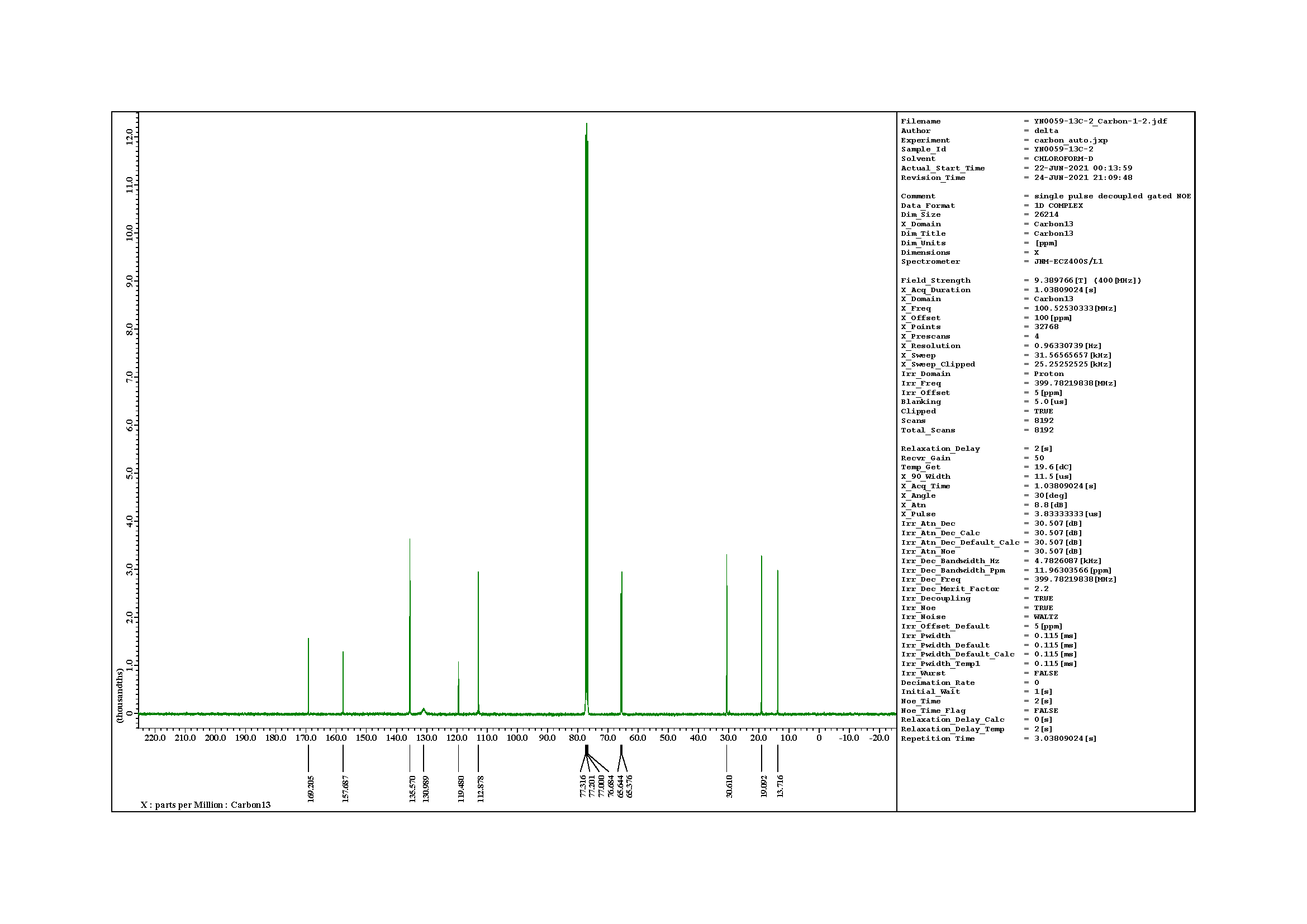


**Supplementary Fig. 21.** ^13^C NMR spectrum of **12** in CDCl_3_ at room temperature.

**Synthesis of *N*-phenyl-4-(10,15,20-triphenylporphyrin-5-yl)benzamide (13)**

5-(4-Carboxyphenyl)-10,15,20-triphenylporphyrin (30.0 mg, 0.046 mmol), thionyl chloride (66.1 μL, 108.3 mg, 0.911 mmol, 20 eq), *N*,*N*’-dimethylformamide (50 μL) were dissolved in THF (1.0 mL) and stirred for 6 hours at room temperature. After the reaction, all volatiles were removed in vacuo. Aniline (12.5 μL, 12.7 mg, 0.137 mmol) in a mixed solvent (THF/CH_2_Cl_2_ = 2 mL /2 mL) was added to the residue, and the reaction mixture was stirred for 12 hours at room temperature. All volatiles were removed in vacuo, and the residue was washed with MeOH repeatedly to give **13** as a purple solid (9.6 mg, 30%).

^1^H NMR (CDCl_3_) δ 8.89 (2H, d, *J* = 4.4 Hz), 8.82 (2H, d, *J* = 4.4 Hz), 8.36 (2H, d, *J* = 7.2 Hz), 8.24–8.23 (3H, m), 8.22–8.21 (3H, m), 8.15 (1H, s), 7.83–7.74 (11H, m), 7.48 (2H, t, *J* = 8.0 Hz), 7.24 (1H, d, *J* = 7.6 Hz), –2.76 (2H, s). ^13^C-NMR (CDCl_3_) δ 165.8, 146.0, 142.0, 138.0, 134.9, 134.5, 134.3, 131.3 (br s, α and β positions of the pyrroles, and the *meso* positions of the porphyrin ring were overlapped), 129.3, 127.8, 126.7, 125.4, 124.8, 120.6, 120.4, 120.3, 118.3. FT-IR (ATR, ZnSe) 3317, 3056, 3029, 1809, 1645, 1598, 1540, 1498, 1473, 1440, 1400, 1349, 1323, 1256, 1221, 1175, 1154, 1104, 1072, 1032, 1021, 1001, 981, 965, 902, 877, 863, 819, 795, 752, 722, 698, 665, 657 cm^–1^. HRMS (ESI) m/z calcd for C_51_H_36_N_5_O (M+H ^+^): 734.29199, found: 734.29184.


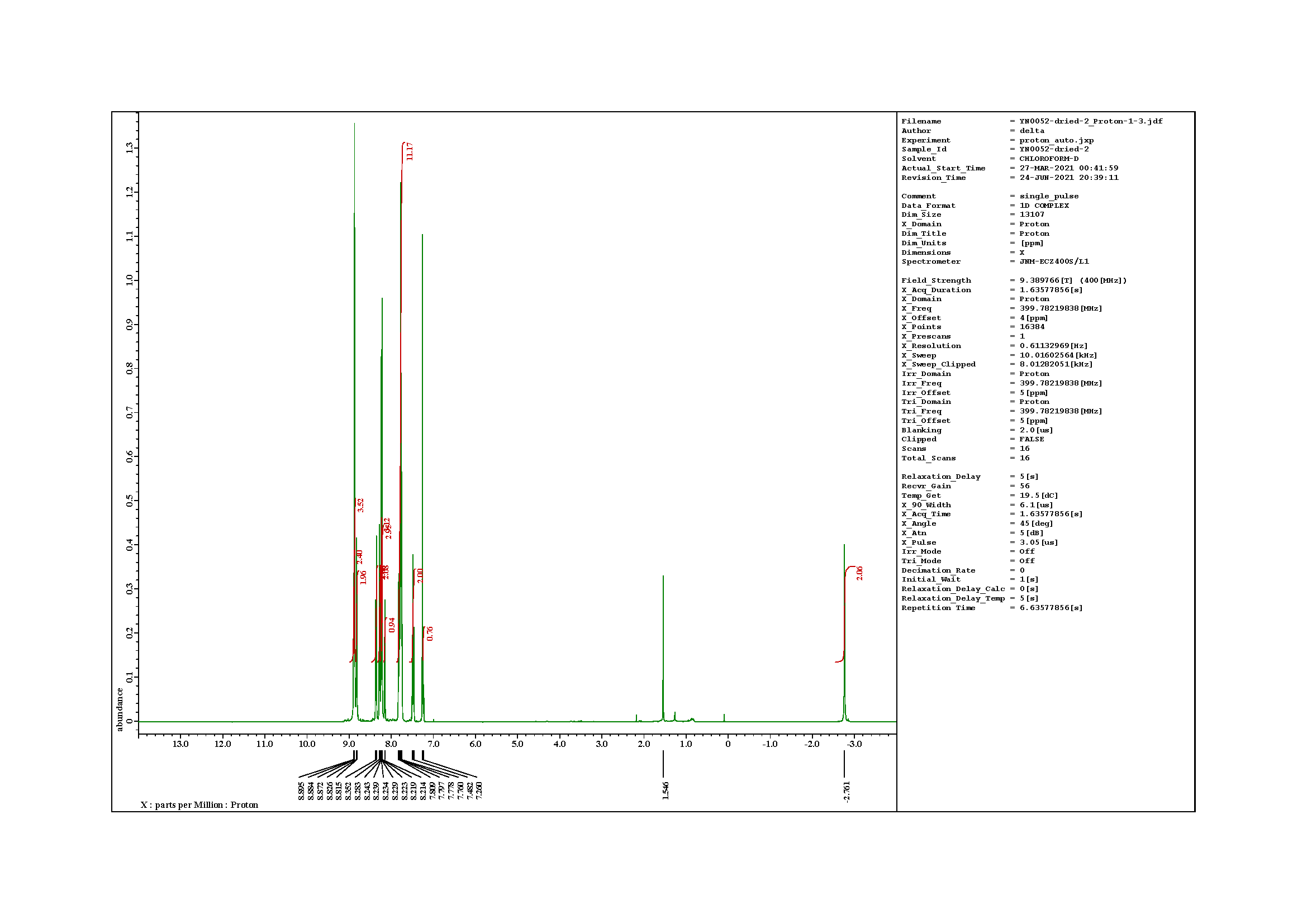
 **Supplementary Fig. 22.** ^1^H NMR spectrum of **13** in CDCl_3_ at room temperature.


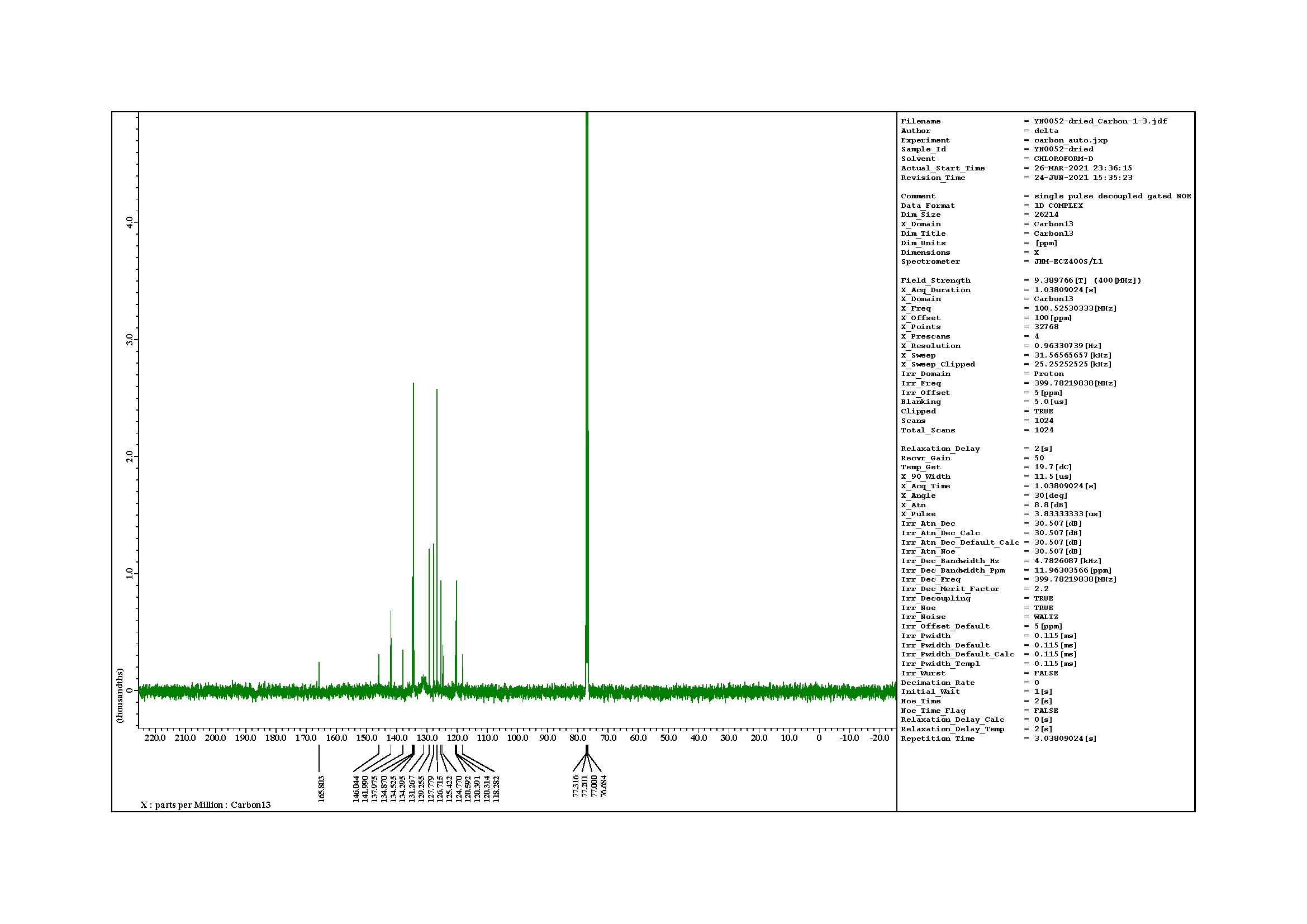
 **Supplementary Fig. 23.** ^13^C NMR spectrum of **13** in CDCl_3_ at room temperature.

**Synthesis of tetrapentyl 4,4',4'',4'''-(porphyrin-5,10,15,20-tetrayl)tetrabenzoate (14)**

Tetrakis(4-carboxyphenyl)porphyrin (50.0 mg, 0.063 mmol), 1-(3-dimethylaminopropyl)-3-ethylcarbodiimide hydrochloride (121.2 mg, 0.632 mmol, 10 eq), 4-dimethylaminopyridine (77.2 mg, 0.632 mmol, 10 eq), and 1-pentanol (136.9 μL, 0.112 mg, 1.265 mmol, 20 eq) were dissolved in tetrahydrofuran (2.5 mL) and stirred for 18 hours at 40 °C. After the reaction, all volatiles were removed in vacuo and the residue was diluted with CHCl_3_ (5 mL) and washed with water (5 mL). The organic layer was dried with Na_2_SO_4_, and then all volatiles were removed in vacuo. The residue was purified with silica gel flash column chromatography (*n*-hexane/AcOEt = 75/25) to give **14** as a purple solid (32.5 mg, 48%).

^1^H-NMR (CDCl_3_) δ 8.47 (8H, d, *J* = 8.8 Hz), 8.31 (8H, d, *J* = 8.0 Hz), 4.52 (8H, t, *J* = 6.8 Hz), 1.97–1.90 (8H, m), 1.61–1.45 (16H, m), 1.02 (12H, t, *J* = 7.2 Hz), –2.80 (2H, s). ^13^C-NMR (CDCl_3_) δ 166.8, 146.5, 134.5, 131.2, 130.1 (br s, α and β positions of pyrrole, and the *meso* position of the porphyrin ring were overlapped), 127.9, 119.4, 106.3, 65.5, 28.6, 28.3, 22.5, 14.1. FT-IR (ATR, ZnSe) 3440, 3417, 3313, 3114, 3064, 2954, 2927, 2857, 2710, 2613, 2534, 1941, 1709, 1604, 1557, 1505, 1466, 1401, 1348, 1307, 1265, 1224, 1174, 1110, 1095, 1046, 1020, 993, 981, 961, 865, 820, 797, 759, 712 cm^–1^. HRMS (ESI) m/z calcd for C_68_H_70_N_4_O_8_Na (M+Na ^+^): 1093.50913, found: 1093.51267.


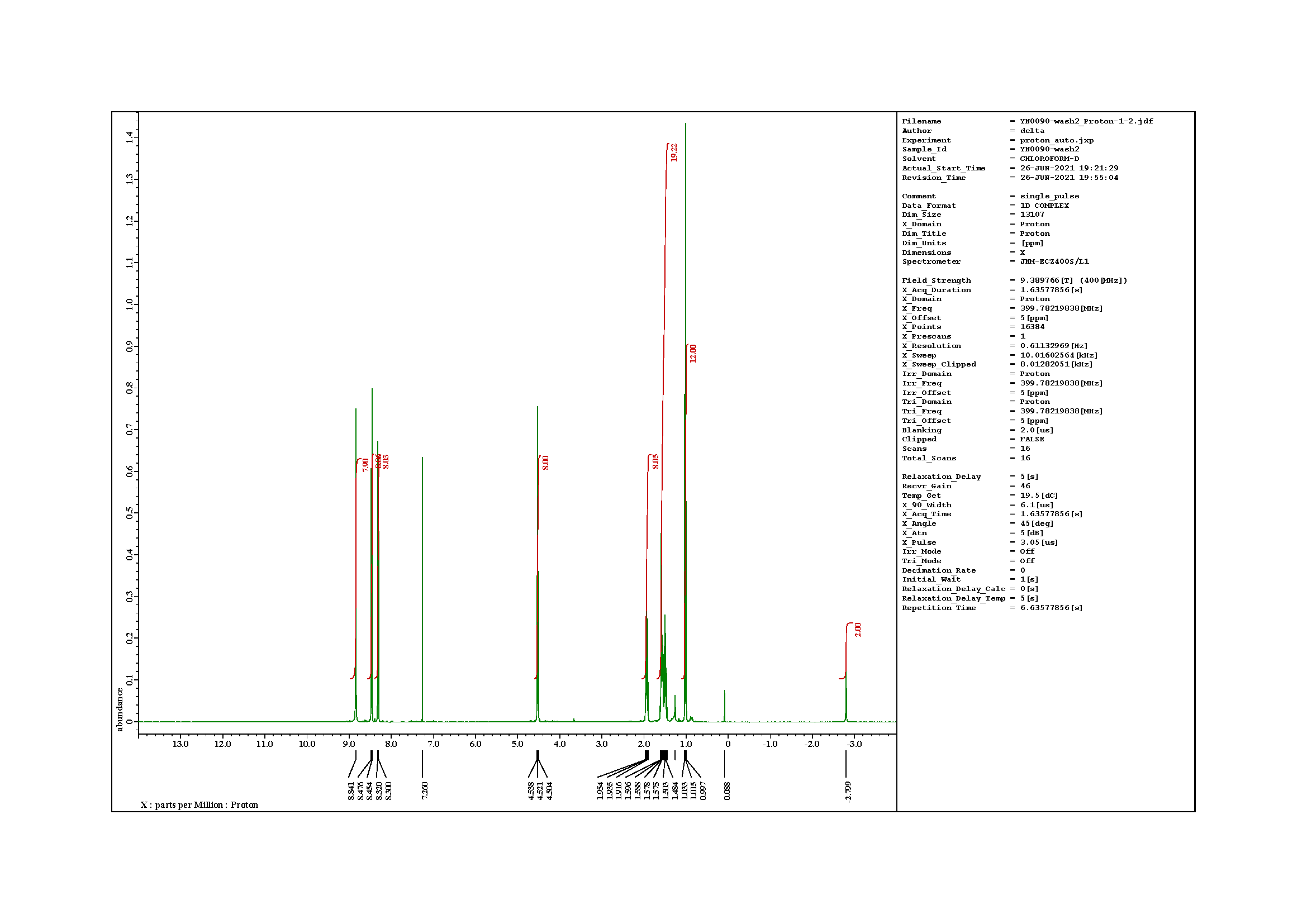


**Supplementary Fig. 24.** ^1^H NMR spectrum of **14** in CDCl_3_ at room temperature.


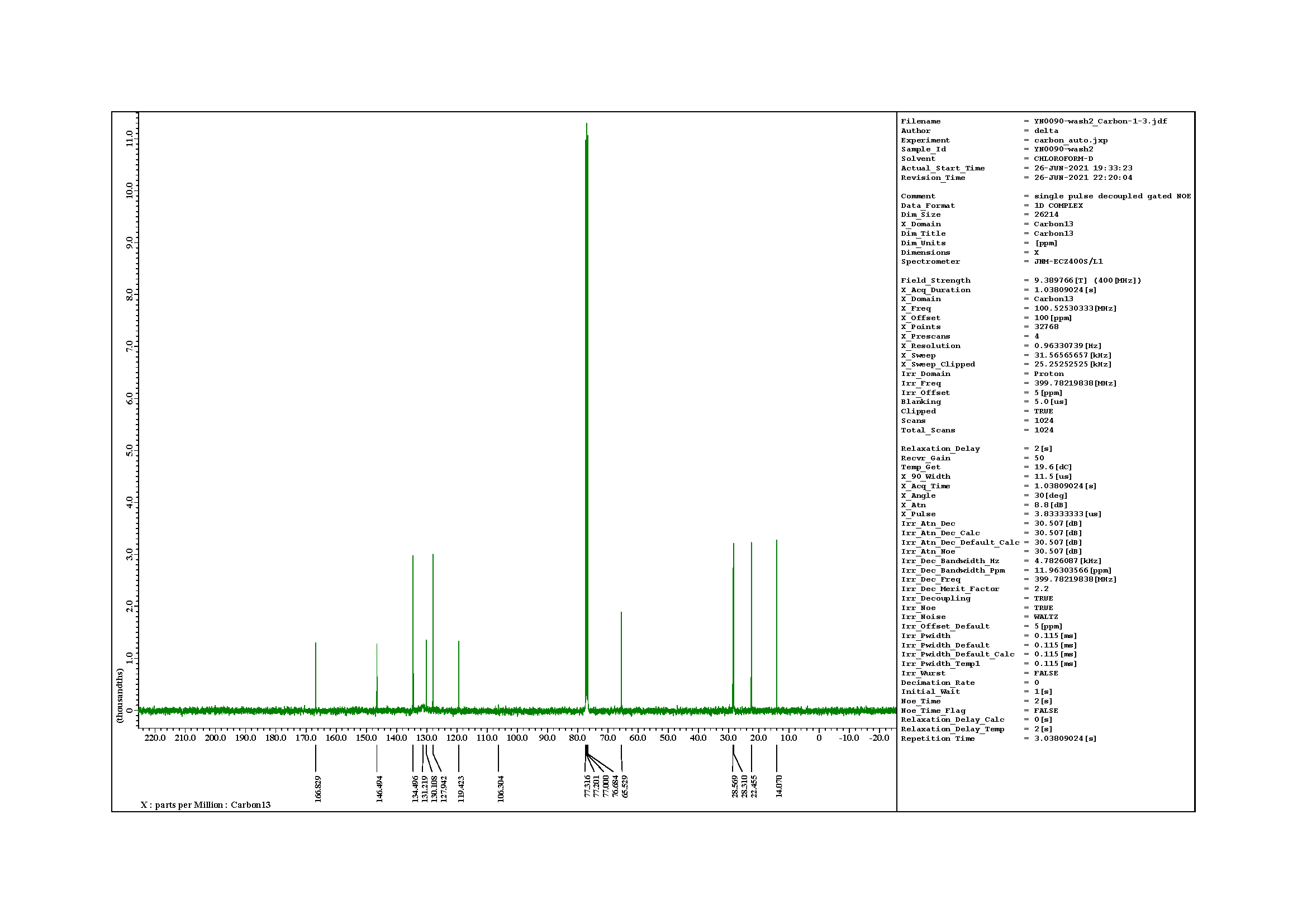


**Supplementary Fig. 25.** ^13^C NMR spectrum of **14** in CDCl_3_ at room temperature.

**Synthesis of 2,2',2'',2'''-((porphyrin-5,10,15,20-tetrayltetrakis(benzene-4,1-diyl))tetrakis(oxy))
tetrakis(*N*-(p-tolyl)acetamide) (15)**

5,10,15,20-Tetrakis(4-carboxymethyloxyphenyl)porphyrin (40.0 mg, 0.061 mmol), 1-(3-dimethylaminopropyl)-3-ethylcarbodiimide hydrochloride (58.2 mg, 0.304 mmol, 5 eq), 4-dimethylaminopyridine (37.1 mg, 0.304 mmol, 5 eq), and *p*-toluidine (55.6 μL, 45.0 mg, 0.607 mmol, 10 eq) were dissolved in *N*,*N*’-dimethylformamide (1.2 mL) and stirred for 36 hours at room temperature. After the reaction, all volatiles were removed in vacuo. The residue was purified with silica gel flash column chromatography (*n*-hexane/AcOEt = 75/25) to give **15** as a dark purple solid (3.8 mg, 5%).

^1^H-NMR (CDCl_3_) δ 8.85 (s, 8H), 8.46 (s, 4H), 8.19 (d, *J* = 8.6 Hz, 8H), 7.60 (d, *J* = 7.9 Hz, 8H), 7.39 (d, *J* = 8.6 Hz, 8H), 7.24 (d, *J* = 7.9 Hz, 8H), 4.92 (s, 8H), 2.38 (s, 12H), –2.79 (s, 2H). ^13^C-NMR (CDCl_3_) δ 166.0, 156.9, 136.3, 135.8, 134.8, 134.3, 129.7, 120.3, 113.2, 67.9, 21.0. The peaks of α and β positions of pyrrole, and the *meso* position of the porphyrin ring could not observed due to the low solubility of this compound. FT-IR (ATR, ZnSe) 3400, 3308, 3029, 2920, 2852, 2534, 1902, 1693, 1603, 1529, 1504, 1471, 1441, 1406, 1351, 1315, 1285, 1225, 1172, 1120, 1108, 1065, 1014, 991, 983, 966, 917, 879, 865, 844, 800, 790, 747, 732, 707, 665 cm^–1^. HRMS (ESI) m/z calcd for C80H67N8O8 (M+H ^+^): 1267.50819, found: 1267.50935.


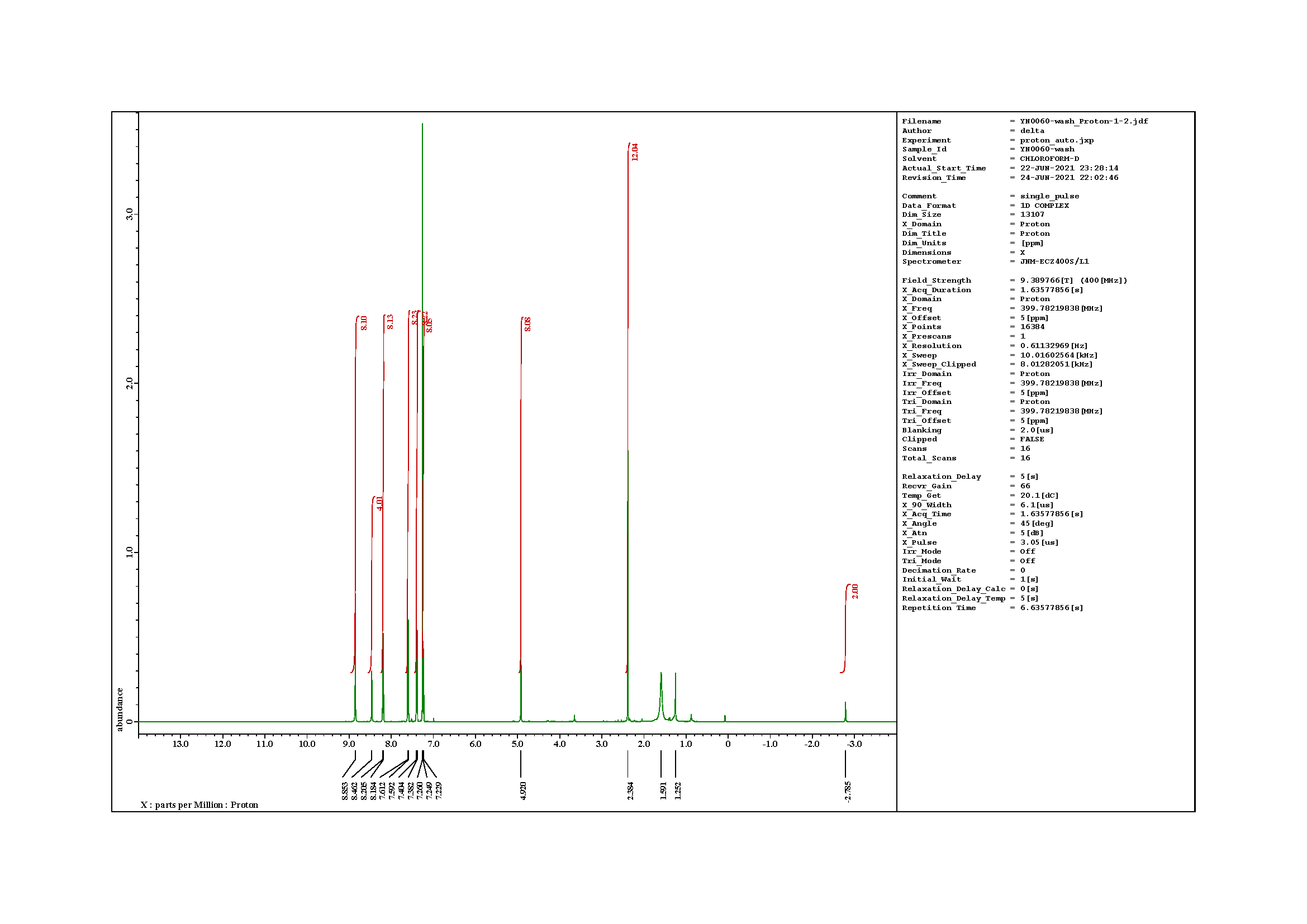


**Supplementary Fig. 26.** ^1^H NMR spectrum of **15** in CDCl_3_ at room temperature.


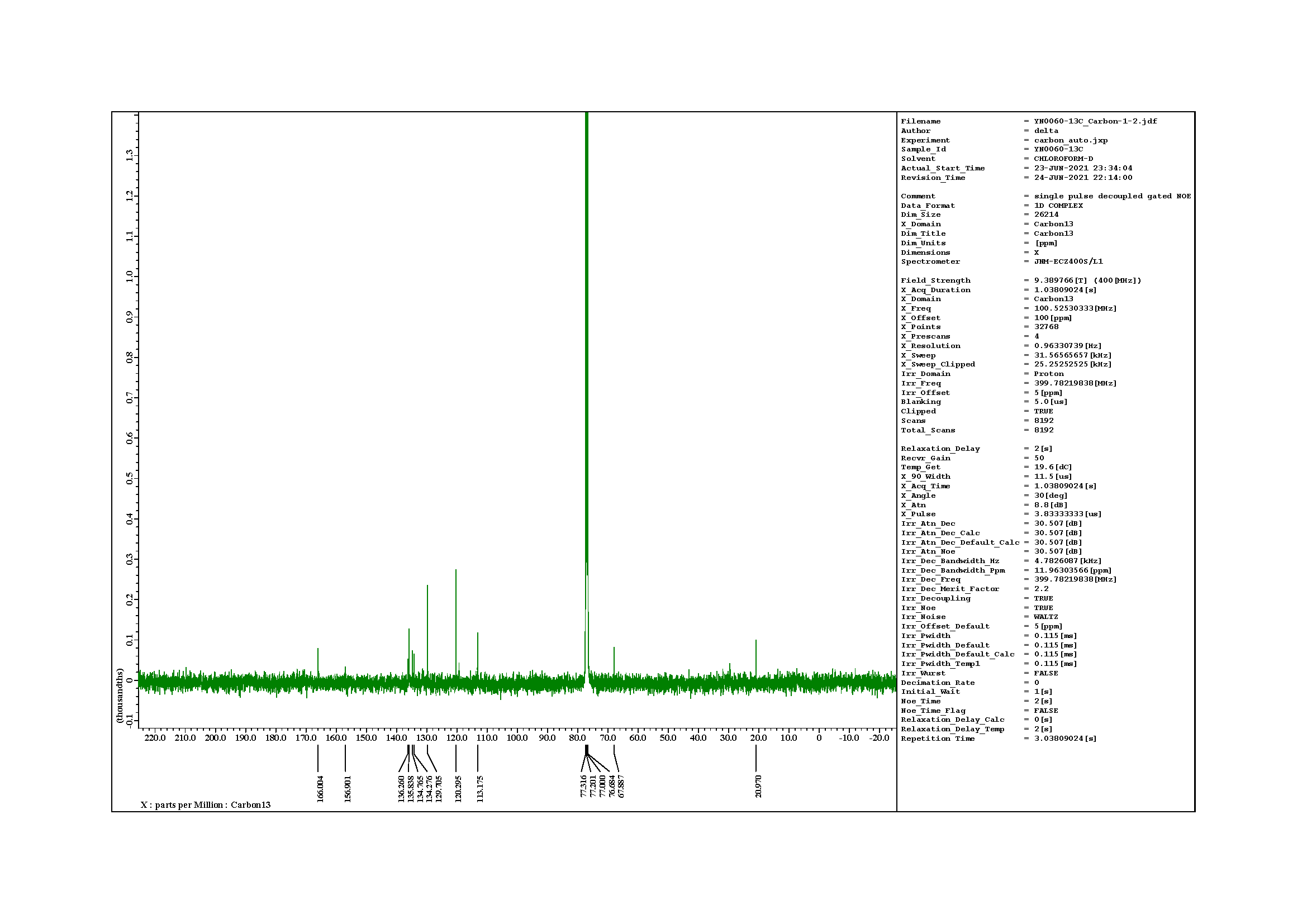
 **Supplementary Fig. 27.** ^13^C NMR spectrum of **15** in CDCl_3_ at room temperature.

Supplementary References

[1] Frisch, M. J. et al. *Gaussian16 Revision C.01*.

[2] Pedregosa, F. et al. Scikit-learn: Machine learning in Python, *the Journal of machine Learning research* **12**, 2825-2830 (2011).
