# Supplementary material for: A semi-automated material exploration scheme to predict the solubilities of tetraphenylporphyrin derivatives
Source: Commun Chem. 2022 Nov 22;5:158. doi: 10.1038/s42004-022-00770-9 (PMC9814751; doi:10.1038/s42004-022-00770-9)
Supplement: Supplementary file 2 — Description of Additional Supplementary Files [file 42004_2022_770_MOESM2_ESM.pdf]

# Description of Additional Supplementary Files

**File name:** Supplementary Data 1

**Description**:  A semi-automated material exploration scheme to predict the solubilities of tetraphenylporphyrin derivatives
